# Current Status and Evolution of Immunotherapy in Glioma Management

**DOI:** 10.7150/ijms.132708

**Published:** 2026-05-01

**Authors:** Jiaying Liu, Guangzhao Yang, Zhengcong Cao, Haozhe Qin, Maorong Zhu, Cheng Zou, Xiao Liu, Yalong He, Jintao Gu

**Affiliations:** 1 Basic Medical College, The Fourth Military Medical University, Xi'an, 710000, China.; 2 State Key Laboratory of Cancer Biology, Biotechnology Center, School of Pharmacy, The Fourth Military Medical University, Xi'an, 710000, China.; 3 Department of Neurosurgery, Xijing Hospital, Xi'an, 710000, China.

**Keywords:** glioma, immunotherapy, immune checkpoint inhibitors, CAR-T cell therapy, cancer vaccine, oncolytic virus

## Abstract

Gliomas, especially glioblastoma (GBM), are the most aggressive primary brain tumors, and their treatment faces multiple challenges. These challenges include incomplete surgical resection, the blood-brain barrier (BBB) that limits drug delivery, and an immunosuppressive tumor microenvironment (TME). Despite the "cold tumor" nature of gliomas, significant progress has been made in immunotherapy strategies. In this review, we focus on the following four strategies: 1) Cancer vaccines, especially personalized vaccines based on neoantigens, can activate specific immune responses; 2) Immune checkpoint inhibitors (ICIs) to reverse T cell exhaustion (e.g., anti-PD-1/PD-L1 antibodies); 3) Adoptive immune cell therapies, particularly chimeric antigen receptor T-cell (CAR-T) therapy, have shown encouraging results in preclinical trials; 4) Oncolytic viruses, which have dual roles of directly lysing tumor cells and stimulating immune responses. Immunotherapy offers a new paradigm to overcome the limitations of conventional glioma therapy. However, tumor heterogeneity, BBB limitation, target selection, and immunosuppressive microenvironment remain major obstacles. Future research should focus on developing combination therapies such as immunotherapy combined with radiotherapy, targeted therapy, exploring novel targets, and using biomarkers for patient stratification, which may help improve the survival outcomes of glioma patients. This review comprehensively evaluates the latest clinical progress of glioma immunotherapy, assesses the current limitations, and explores future directions.

## Introduction

Glioma is a central nervous system tumor arising from glial cells. It is the most common and most aggressive primary brain tumor in adults 1. The pathogenesis of glioma is complex, involving various genetic and molecular alterations 1. The 2021 WHO Classification (5th edition) has shifted the classification of adult-type diffuse gliomas from a purely histologic grading system to an integrated paradigm that combines histologic features with molecular biomarkers 2. These tumors are primarily categorized into three subtypes: 1) IDH-mutant astrocytoma: cases with CDKN2A/B homozygous deletion are directly assigned WHO grade 4, irrespective of classic histologic features such as microvascular proliferation or necrosis 3; 2) IDH-mutant oligodendroglioma with 1p/19q codeletion 3; 3) IDH-wildtype glioblastoma, which is strictly defined as IDH-wildtype grade 4 astrocytoma and represents the most malignant and aggressive subtype 2. Furthermore, distinct genetic alterations are increasingly recognized to shape the tumor immune microenvironment and consequently modulate the response to immunotherapeutic interventions 4. Although its incidence is relatively low, the median survival for GBM patients is only approximately 15 months, indicating a very poor prognosis 5.

Standard treatment includes surgical resection, radiotherapy, and temozolomide (TMZ) chemotherapy 6. Additional therapeutic options include DNA damage response (DDR) inhibitors and targeted molecular therapies 7. Despite these measures, patient outcomes remain poor, with high recurrence rates 7.

The poor prognosis stems from multiple factors. First, the highly invasive nature of glioma cells enables diffuse infiltration into normal brain tissue, precluding complete surgical resection 8. Second, the BBB severely hinders effective drug delivery, limiting systemic therapy efficacy 5. Third, the glioma microenvironment is highly immunosuppressive. It features abundant M2-polarized tumor-associated macrophages (TAMs), increased infiltration of regulatory T cells (Tregs) and myeloid-derived suppressor cells (MDSCs), exhausted cytotoxic T lymphocytes, and upregulation of multiple immune checkpoints 1. These factors, together with metabolic suppression (e.g., tryptophan catabolism via the IDO pathway) and hypoxia-driven immunosuppression, collectively shape the "cold tumor" phenotype of gliomas 9. Furthermore, glioma has a relatively low tumor mutational burden and high intra- and inter-tumoral heterogeneity. This further weakens the immune system's ability to recognize and eliminate the tumor 1. Together, these factors present unique challenges, prompting the exploration of immunotherapy as a new strategy.

Despite inherent immunosuppression, immunotherapy for glioma is feasible 10. Recent re-evaluation of central nervous system (CNS) immune privilege and the discovery of skull-meningeal channels and intracranial lymphatic vessels provide new perspectives on brain tumor immunology 10. Studies show that inducing immunogenic cell death (ICD) can transform the glioma microenvironment from "cold" to "hot", promote Th17 cell migration to the tumor, and enhance immunotherapy efficacy 11. Additionally, immune cell infiltration patterns in the TME correlate with patient prognosis. Stronger immune cell infiltration often predicts better survival 11. These findings provide a theoretical basis for developing immunotherapies for glioma.

Current glioma immunotherapy mainly includes four classic strategies. Cancer vaccines introduce tumor-specific dendritic cells (DCs) or tumor-associated antigens (TAAs) to activate anti-tumor immune responses 12. Personalized vaccines, especially those targeting neoantigens, show great potential 1. ICIs like PD-1, PD-L1, and CTLA-4 inhibitors aim to reverse T cell exhaustion and reactivate anti-tumor immunity 1. Based on the available literature, adoptive immune cell therapy has not yet demonstrated satisfactory clinical efficacy in glioma studies, and its safety profile requires further investigation 13-15. However, breakthroughs in new therapies such as universal CAR-T, fourth-generation CAR-T, and novel-target CAR-T in preclinical research have brought hope for improving the prognosis of glioma patients. Oncolytic viruses have a dual mechanism: directly killing tumor cells and activating immune responses. They have made breakthrough progress in clinical trials 1. This review comprehensively evaluates recent clinical progress in these four main immunotherapy strategies for glioma. It critically analyzes limitations and provides insights into future directions.

## 1. Cancer Vaccines

**Figure 1:**
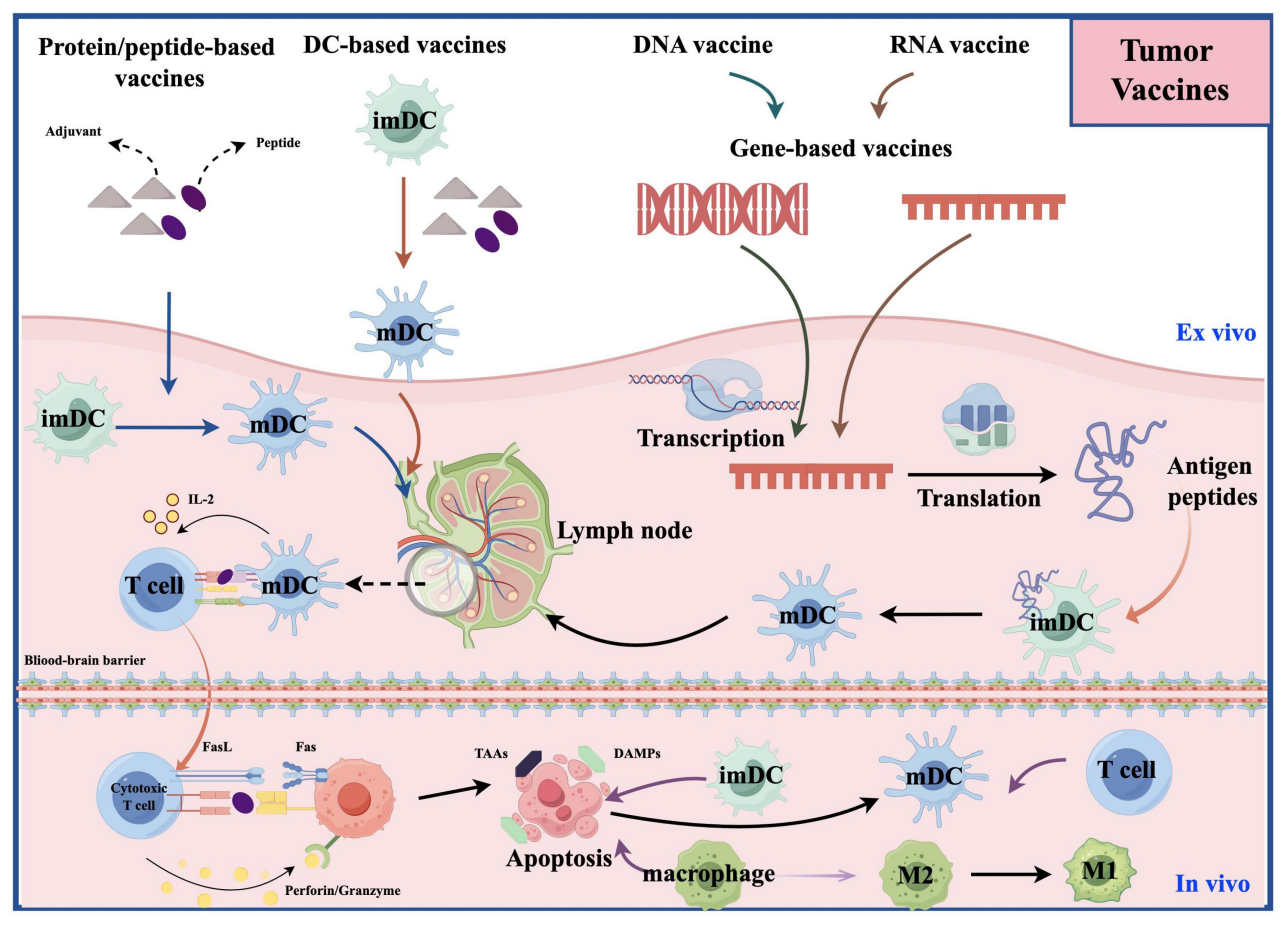
The figure illustrates the mechanisms of action of four types of vaccines: dendritic cell vaccines, peptide vaccines, DNA vaccines, and RNA vaccines. These vaccines promote the activation and maturation of immune cells by presenting tumor-associated antigens. Subsequently, the activated immune cells induce immunogenic cell death of tumor cells, which in turn leads to the release of a broader repertoire of tumor-associated antigens and neoantigens. The newly released antigens further recruit and activate additional immune cells and also promote the reversion of suppressive immune cells, thereby effectively reversing the immunosuppressive state within the TME.

Cancer vaccines aim to induce the immune system to recognize and attack TAAs or tumor-specific antigens (TSAs) 12 **(Figure 1)**. In early exploration (around the 1990s), the main type of tumor vaccine was the whole tumor cell vaccine 16. They had manageable safety but limited clinical efficacy 16; In the early 2000s, DC vaccines emerged. They are made by isolating monocytes from patients' blood, differentiating them into DCs *in vitro*, loading them with tumor antigens (e.g., cell lysates, synthetic peptides), and reinfusing them to activate specific T cells 17. In a large Phase 3 trial of a dendritic cell vaccine, the median overall survival (OS) among 331 patients was 23.1 months. Among the 131 patients with MGMT promoter methylation, the 3-year survival rate reached 46.4%. These findings provide strong evidence supporting the survival benefit of the DC vaccine 16; Around the 2010s, molecularly targeted peptide vaccines appeared. They use defined immunodominant epitope peptides to induce precise immune responses 18. IMA950 is the first multi-peptide vaccine targeting glioblastoma to enter clinical trials 19. In its first-in-human Phase I study, it successfully met the safety and immunogenicity endpoints, marking a transition in glioma vaccine development from cell-based approaches to precision molecular targeting 19. Since 2015, personalized/neoantigen vaccines have developed rapidly. These are peptide-based personalized cancer vaccines composed of immunogenic mutant epitopes derived from the patient's specific glioma 20.

### 1.1 Dendritic Cell Vaccines

Based on literature from 2015-2025, 14 DC vaccine studies entered clinical trials. Comparative analysis preliminarily confirmed the safety and tolerability of DC vaccines 21-25, demonstrating good prospects in the field of glioma immunotherapy. Representative studies include 1) Wilms Tumor 1 (WT1) mRNA vaccine 21; 2) autologous tumor antigen (ATA) vaccine 23, 24; 3) ICT-107 25; 4) DC vaccines loaded with glioma stem cell (GSC) (A2B5^+^) antigen 26; 5) DC vaccines targeting CMVpp65 27-29. First, in a single-arm Phase I/II study of advanced solid tumors, WT1 mRNA-electroporated autologous DCs (WT1-mRNA/DC) demonstrated promising therapeutic activity. The induced type 1 T-cell responses were associated with improved OS 21. Second, the ATA vaccine's antigens come from self-renewing irradiated autologous tumor cells in culture. It contains personal neoantigens and any GBM TAAs, but not normal cell antigens, offering potential advantages over autologous tumor lysate 23. However, its production requires sufficient tumor tissue, limiting use in advanced patients 23. Finally, the efficacy and safety of ICT-107 and GSC antigen-loaded DC vaccines need further validation in larger trials 25, 26.

Specific pretreatment of DC vaccines (e.g., using tetanus-diphtheria (Td) toxoid 28, 29) combined with adjuvants (e.g., keyhole limpet hemocyanin, KLH) 21 or cytokines (e.g., GM-CSF) 25, or using activators (e.g., IFNγ or lipopolysaccharide 25), can enhance vaccine potency and patient immune responses to TSAs. This may improve patient outcomes 21, 25. Many studies indicate that patients with O-6-methylguanine-DNA methyltransferase (MGMT) promoter methylation, combined Telomerase Reverse Transcriptase (TERT) promoter and Isocitrate Dehydrogenase 1/2 (IDH1/2) mutations, low B7-H4 expression, Human Leukocyte Antigen A2 (HLA-A2) positivity, and high immune responses are more sensitive to DC vaccines. This may be associated with better prognosis 25, 26, 30.

### 1.2 Molecularly Targeted Peptide Vaccines

Representative studies from 2015 to 2025 include 1) the Epidermal Growth Factor Receptor variant III (EGFRvIII) targeted vaccine (Rindopepimut/CDX-110) 31, 32; 2) IDH mutation-targeted vaccine (IDH1-vac) 33; 3) Survivin-targeting peptide vaccine (SurVaxM) 34, 35; 4) and WT1 peptide vaccine 36 as single-target vaccines. Multi-target peptide vaccines include 1) IMA950 19, 37; 2) WT1 peptide cocktail vaccine 38; 3) VEGFR1-VEGFR2 peptide vaccine 39; 4) glioma-associated antigen (GAA) synthetic peptide vaccine 40.

#### 1.2.1 Single-Target Vaccines

First, Rindopepimut is a single-target peptide vaccine against EGFRvIII. It consists of an EGFRvIII-specific peptide conjugated to KLH. In the ACT III trial, Rindopepimut successfully induced specific immune responses, achieving a 5.5-month PFS rate of 66%, significantly exceeding the preset null hypothesis of 53% (P=0.0168) 32. In terms of safety, vaccine-related adverse events were common but generally tolerable 32. However, the subsequent Phase III ACT IV trial showed that Rindopepimut did not improve survival in patients with newly diagnosed glioblastoma (nGBM) 31. The ACT III trial demonstrated promising efficacy; however, the subsequent Phase III ACT IV trial failed to confirm these findings. Several factors may account for this discrepancy: 1) Patient selection bias. ACT III enrolled a highly selected population with favorable responses to initial therapy 32. Comparing their survival outcomes with unselected historical controls likely overestimated the therapeutic benefit of the vaccine 32. 2) Improvements in standard of care. In ACT IV, the median OS in the Rindopepimut arm (20.1 months) was consistent with that observed in ACT III, indicating stable vaccine activity 31. However, advances in standard therapy likely prolonged survival in the control group relative to historical data, thereby diluting the relative benefit of Rindopepimut and rendering the difference statistically nonsignificant 31. 3) Instability of target expression. In a small subset of ACT IV patients, EGFRvIII expression was lost at recurrence in approximately 60% of cases, irrespective of treatment received 31. This finding suggests that single-target strategies against EGFRvIII may be limited and supports the exploration of multi-target approaches in future studies 31. Second, the IDH1(R132H) peptide vaccine (IDH1-vac) showed good safety in its first trial, but the pseudoprogression rate was high, correlating with increased peripheral T cell responses 33. Third, SurVaxM has good safety and tolerability 34, 41. Clinically, benefit was observed in both methylated and unmethylated patients, with longer survival in the former. Notably, this clinical benefit was associated with upregulation of tumor-associated B cells, which predicted better outcomes 35. However, it is essential to interpret such early-phase findings with caution. A well-documented challenge in GBM research is the frequent discrepancy between encouraging Phase I/II results and the outcomes of subsequent Phase III trials. Initial efficacy signals often fail to be reproduced in larger, more rigorous, and controlled settings, as exemplified by the Rindopepimut trials and other candidate immunotherapies in neuro-oncology 31, 32, 42, 43. A large randomized trial of SurVaxM for nGBM (NCT02455557) was completed in March 2026, but the results have not yet been reported. Finally, the WT1 peptide (WT2725) vaccine is safe and tolerable but its efficacy needs confirmation 36.

#### 1.2.2 Multi-Target Vaccines

First, IMA950 is a novel multi-target vaccine containing 11 tumor-associated peptides identified from HLA surface receptors in GBM tissue. It was used to induce antigen-specific peripheral CD8^+^ T cell responses in HLA-A*02-positive patients. In terms of safety, drug-related toxicity was relatively benign 19. Second, combining TMZ with VEGFR1-VEGFR2 peptide vaccine showed synergistic activity 39. Third, the GAA synthetic peptide vaccine targets IL-13Rα2, EphA2, WT1, and Survivin. The adjuvant poly-ICLC promotes type 1 polarization of T cell responses against these antigens, enabling effective T cell infiltration into brain tumors and therapeutic benefit 40. Furthermore, patients with WHO grade II astrocytoma or oligoastrocytoma without prior non-surgical treatment may be particularly suitable for this vaccine 40. Finally, the WT1 HLA class I/II peptide cocktail vaccine is safe in patients 38.

### 1.3 Personalized/Neoantigen Vaccines

Representative studies from 2015 to 2025 include 1) IDH1 mutation individualized peptide vaccine 20; 2) personalized peptide vaccine (PPV) 44; 3) Glioma Active Personalized Vaccine Consortium (GAPVAC) 45; 4) autologous formalin-fixed tumor vaccine (AFTV) 46. First, a clinical trial of a personalized neoantigen-targeting vaccine for IDH1-mutant glioma used whole-exome sequencing to customize treatment, which can cover tumor heterogeneity 20. Results showed that the vaccine was well-tolerated and induced persistent T cell responses 20. Moreover, patients with T cell responses to multiple neoantigens had a trend toward longer OS compared to those with no/few responses 20. Additionally, detection of CDR3 sequences may help track CD4^+^ and CD8^+^ T cell clones in blood and tumor tissue 20. However, this study was retrospective and non-randomized, with variability in the number of peptides administered and the timing of vaccinations. Therefore, the findings warrant further validation in prospective randomized controlled trials. Second, a PPV trial for HLA-A24-positive recurrent GBM (rGBM) found that SART2-93 positivity was a major factor limiting clinical benefit for all patients 44. In contrast, biomarkers associated with better OS included lower proportions of CD11b^+^CD14^+^HLA-DR^low^ immunosuppressive monocytes, higher proportions of CD4^+^CD45RA^-^ activated T cells, and medium levels of CCL2, VEGF, IL-6, IL-17, and haptoglobin 44. Third, AFTV is a personalized vaccine derived from formalin-fixed autologous glioblastoma specimens 46. Theoretically, it preserves all tumor antigens, including neoantigens, obviating the need for HLA-based patient selection 46. However, this strategy has two major limitations: the inability to monitor therapeutic response via specific antibody titers, and the dependence of vaccine yield on the amount of resected tumor tissue, precluding flexible dose escalation or prolonged administration. A Phase IIb double-blind randomized trial showed no significant difference in efficacy between the vaccine and placebo groups in the overall population, likely due to the small sample size and the high quality of surgery and TMZ therapy received by the placebo group (median OS 31.5 months) 46. Subgroup analyses suggested that patients with gross total resection or p53-negative tumors may be more sensitive to AFTV. This hypothesis is being evaluated in a completed Phase III trial (UMIN000010602), the results of which have not yet been reported 46. Finally, the GAPVAC vaccine consists of APVAC1 (a pre-manufactured unmutated antigen repertoire) and APVAC2 (targeting neoepitopes) for HLA-A*02:01^+^ or HLA-A*24:02^+^ nGBM patients 45. Results showed that APVAC1 induced sustained central memory CD8^+^ T cell responses, while APVAC2 induced Th1-type CD4^+^ T cell responses against predicted neoepitopes 45. Furthermore, both vaccines, administered with poly-ICLC and GM-CSF adjuvants, were considered safe and highly immunogenic 45.

### 1.4 New Preclinical Advances

Recent advances focus on new delivery systems/pathways, optimized antigen selection, immune microenvironment modulation, and personalized vaccine design.

#### 1.4.1 New Delivery Systems

Nanotechnology offers promising solutions. Optimizing particle size, surface modification, and targeting ligands significantly improves brain enrichment and immune activation 47-50. Representative platforms include lipid nanoparticles (LNPs), polymer nanoparticles, and inorganic nanomaterials. A key LNP-based preclinical study is the CVGBM vaccine. CVGBM is a novel multi-targeted mRNA-LNP vaccine encoding a fusion protein comprising eight tumor-associated antigen-derived epitopes 48. Its platform efficacy was validated in a surrogate melanoma mouse model, and antigen presentation specificity was confirmed *in vitro* 48. However, immunopeptidomics analysis detected the presentation of only four of the eight encoded epitopes, while the remaining four were not identified 48. Currently, a Phase I trial for HLA-A*02:01^+^ MGMT unmethylated GBM is ongoing (NCT05938387) 48. Although several mRNA-LNP vaccines have entered clinical trials for infectious diseases and cancer, their clinical translation remains limited by poor targeting precision, potential immunogenicity and toxicity, and the difficulty of developing universal delivery systems 51. Biomimetic nano-DC vaccines show improved biocompatibility, targeting, and immune activation 52. For example, conjugating biomimetic DC membranes with tumor-associated costimulatory molecules or paclitaxel nanoparticles, combined with ICIs, alleviated T cell exhaustion in mouse models, significantly extended survival, potentially overcoming GBM immunotherapy resistance 53, 54. However, the clinical translation of cell membrane-coated nanoparticle technology is hindered by challenges in scalable production and purification, safety assurance, quality control, and analytical method development 52.

#### 1.4.2 Delivery Route Selection and Optimization

Vaccine administration route, frequency, and timing significantly impact efficacy and require systematic optimization. 1) The intranasal route is an innovative strategy 49. It bypasses the BBB, delivering vaccine directly to the brain and cervical lymph nodes 49. It reduces systemic exposure and has good patient compliance 49. Sequential targeted sonodynamic nanovaccine (Stars NV) 55, Survivin peptide-CpG nanovaccines (SPOD-NV) 49, and enhanced nanovaccines using biomineralizing virus-like particles 50 are delivered through the nasal cavity, with drug enrichment observed in the brain and lymph nodes 49, 50, 55. 2) Intravenous delivery is an established technique, allows high dosing, and activates immune cells in systemic lymphoid organs 49. Serum IgG titers were higher after intravenous delivery (IV) than after intranasal delivery 49. However, challenges include poor brain/tumor penetration, rapid clearance by liver macrophages, potential cytokine storm, and systemic toxicity risk 49. A sequential dosing strategy for SPOD-NV vaccine used 5 full IV, 5 full intranasal, or 3 intranasal + 2 IV injections, all combined with anti-CTLA-4 antibody 49. Mice with full IV dosing had significantly longer median survival than full intranasal 49. The 3 intranasal + 2 IV group extended survival to 41 days, with a 43% complete response rate 49. This suggests combining intranasal and IV routes may synergize, producing local and systemic immunity, improving efficacy 49. 3) Subcutaneous delivery allows antigen uptake by subcutaneous DCs, which migrate to regional lymph nodes 49. In the SPOD-NV study, subcutaneous + IV sequential dosing was significantly less effective than intranasal + IV, highlighting the key role of intranasal delivery in GBM treatment 49. Intratumoral delivery directly delivers drugs to the tumor, bypassing the BBB 56. It enables local immune activation, remodels the TME, and reduces systemic exposure risk 56. For example, intratumoral pIpC-Dox LNPs significantly increased infiltration and activation of TAMs, DCs, and T cells. Drawbacks include invasive procedure, difficulty covering infiltrative cells, and increased risk with multiple injections 56.

#### 1.4.3 Optimized Antigen Selection

Neoantigens are antigens produced by specific mutations in tumor cells 20. They are specific and immunogenic, and do not exist in normal tissues, thus becoming ideal vaccine targets 20. Representative studies include the IDH1-R132H 20-mer peptide vaccine 20 and nanoshield vaccines 57. The (multi-cationic protein (MCP))-muIDH1 nanoshield vaccine (NaV) uses a MCP scaffold, genetically fuses the muIDH1 neoantigen to MCP, and assembles carboxylated PEG into 100 nm spherical nanoparticles 57. This enables lysosomal escape, enhanced cross-presentation, NLRP3 inflammasome activation, and prolonged *in vivo* retention 57. In tumor-bearing mice, combining NaV with anti-PD1 delayed postoperative recurrence and increased median survival by over 40% 57.

TAAs are normal proteins overexpressed or over-presented on tumor cells 48, 49, 58, 59. While less immunogenic than neoantigens, they are shared, potentially benefiting more GBM patients 48, 49, 58, 59. Representative TAAs include 1) Survivin Baculoviral IAP Repeat Containing 5 49; 2) Ephrin type-A receptor 2 49; 3) Plasminogen activator urokinase receptor 48; 4) Transforming growth factor beta 2 (TGFB2) 58. Representative vaccines include 1) SurVaxM 49; 2) CVGBM mRNA vaccine 48; 3) and GVac 58. GVac is a multi-epitope vaccine targeting TGFB2, and its antigen selection and screening have been strongly assisted by computer-aided design 58. Allergenicity, antigenicity, toxicity, epitope selection, molecular docking, dynamics simulation, and immune simulation can be predicted computationally, theoretically enabling rapid screening of low-toxicity, highly immunogenic candidate vaccines with strong target affinity, though experimental validation is needed 58.

#### 1.4.4 Immune Microenvironment Modulation

GBM's immunosuppressive TME limits vaccine efficacy 7. New vaccine strategies remodel the TME by reprogramming TAMs, reducing Tregs, and enhancing T cell infiltration 47, 49, 55, 57, 60. However, the observed TAM reprogramming is mediated not by the vaccine itself, but by secondary components such as sonosensitizers 55 or ICIs 61 incorporated into these strategies. The vaccine Star NV contains a sonosensitizer, the Toll-like receptor agonist R848 (which promotes M1 polarization), and CM-β-CD (for macrophage targeting) 55. Mechanically, it uses ultrasound to activate sonosensitizers, generate reactive oxygen species (ROS) in tumor tissues to directly destroy GBM cells, and release R848 to achieve TAM transformation to M1 phenotype 55. *In vivo* experiments in the glioma mouse model confirmed that this method could kill tumor cells and help to reshape the immunosuppressive microenvironment 55. Clofazimine (CFZ), an anti-leprosy drug, has potent Wnt/β-catenin inhibitory activity 60. Combining DC vaccine with CFZ inhibited the Wnt/β-catenin pathway in cancer stem cells, blocking glioma phenotypic heterogeneity/plasticity and β-catenin-mediated immune escape, thereby reducing immunosuppression 60. Immune checkpoint synergy can also improve the TME. For example, nanoshield vaccine combined with anti-PD-1 improved postoperative relapse-free survival by over 40% in mouse models 57.

## 2. Immune Checkpoint Inhibitors

**Figure 2:**
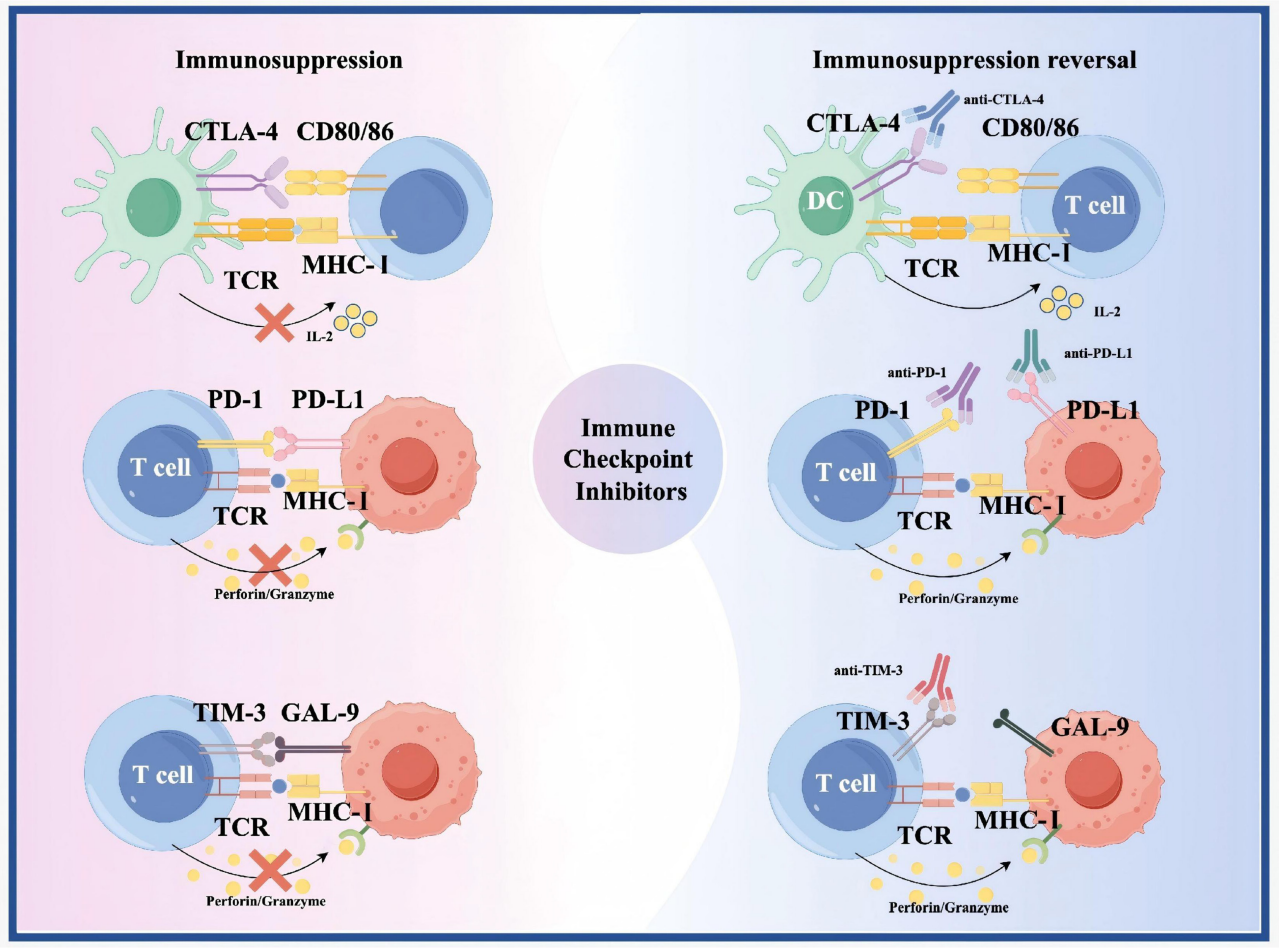
As shown in the figure, immune checkpoint molecules such as CTLA-4, PD-1, PD-L1, and TIM-3 negatively regulate T cell activation and T cell-mediated tumor cell killing. Immune checkpoint inhibitors block these inhibitory signaling pathways, relieve T cell exhaustion, and subsequently restore the capacity of T cells to recognize and clear tumor cells.

Immune checkpoint molecules limit autoimmunity by regulating T cell responses to self-antigens 62. However, they also limit attacks on cancer cells 62. ICIs block these molecules, enhancing T cell recognition and killing of cancer cells 62 **(Figure 2)**. Between 1980 and 2000, key immune checkpoints, including CTLA-4 and PD-1 were identified, establishing the foundation for ICIs 63, 64. In 1996, Allison and colleagues first demonstrated that CTLA-4 blockade enhances antitumor immunity in preclinical models 65. PD-1 was cloned by Honjo's team in 1992, and its role as an immune checkpoint was elucidated by 2000 63. Following regulatory approval of the PD-1 inhibitors Nivolumab and Pembrolizumab in 2014, clinical trials in glioma were initiated. The first-in-human Phase I study of Nivolumab in recurrent glioblastoma (CheckMate 143) established its safety and tolerability 66. However, the subsequent Phase III CheckMate 143 trial failed to demonstrate an OS benefit for Nivolumab over Bevacizumab in recurrent glioblastoma, underscoring the limitations of ICI monotherapy in this disease 67. After 2015, ICI trials and combination therapies for glioma increased. According to the literature, ICIs used in trials in the past 10 years include: 1) anti-CTLA-4 (Ipilimumab) 42, 43, 68; 2) anti-PD-1 (Nivolumab, Pembrolizumab) 42, 69; 3) anti-PD-L1 (Atezolizumab) 70. The efficacy of ICI monotherapy is limited, and clinical studies often combine multiple ICIs or other drugs or as adjuvant therapy 71.

### 2.1 Clinical Trials and Combination Therapies

Representative clinical studies from the past decade include: 1) Ipilimumab + Nivolumab for nGBM (two studies) 42, 43; 2) Atezolizumab + Radiotherapy + TMZ Phase I/II trial (NCT03174197) 70; 3) Evaluation of neoadjuvant Pembrolizumab in rGBM 69; 4) Nivolumab + Ipilimumab before radiotherapy for nGBM Phase I trial 68; 5) Bevacizumab + anti-PD-1 therapy (NCT05502991, NCT05540275) 72, 73.

The safety and efficacy of Ipilimumab + Nivolumab for nGBM were demonstrated in the NRG Oncology BN002 (Phase I) trial 42. However, the randomized Phase II/III NRG Oncology BN007 trial for MGMT-unmethylated nGBM found that Ipilimumab + Nivolumab did not improve Progression-Free Survival (PFS) compared to TMZ 43. Subsequently, multiple clinical trials have been completed, and despite the use of various immune checkpoint inhibitor regimens, none have demonstrated superiority of ICI monotherapy or combination therapy over standard treatment 43, 74, 75. Thus, without molecular selection or biomarker guidance, ICIs confer extremely limited survival benefit 43. Nonetheless, the identification of patients with specific genetic features or immune phenotypes sensitive to ICIs remains of great value, and relevant biomarker analyses are currently ongoing 43. Atezolizumab + Radiotherapy + TMZ was well-tolerated in a Phase I/II trial (NCT03174197). Unlike DC vaccine sensitivity, this therapy was more effective in PTEN-mutant, mesenchymal subtype GBM patients, less effective in EGFR-mutant, proneural subtype, and significantly better in MGMT-methylated vs. unmethylated patients 70. Additionally, GBM immune enrichment correlated highly with pre-treatment tumor mesenchymal subtype and patient gut microbiota 70. Moreover, longer-surviving patients had enriched Bacillota phylum bacteria in stool samples 70. A trial of neoadjuvant Pembrolizumab in rGBM showed that treatment activity correlated with shortened/reduced cell cycle and proliferation genes and increased T cell/interferon-related genes 69. This suggests that combined transcription factors that reduce cell cycle/proliferation gene expression or increase T cell/interferon gene expression may improve efficacy 69. Also, ICIs may have inherent resistance due to chronic inflammation in the TME, suggesting combining anti-inflammatory drugs might restore neoadjuvant ICI activity 69. Phase I clinical trials of Nivolumab + Ipilimumab in the treatment of nGBM before radiotherapy found that advanced tumors have high LAG-3 levels, downregulation of PPAR signaling and up-regulation of TGF-β and ERBB signaling, and dysregulation of these pathways is associated with ICI resistance 68. This suggests combining LAG-3 inhibitors, PPAR agonists, and TGF-β/ERBB pathway inhibitors might overcome resistance and improve efficacy 68. Two studies (NCT05502991, NCT05540275) found that Bevacizumab plus anti-PD-1 significantly improved OS in patients with rGBM. Furthermore, tumor *in situ* fluid circulating tumor DNA technology can monitor therapeutic response and guide therapy 72, 73. Collectively, these findings are promising, suggesting pre-treatment screening for patients likely to respond to Bevacizumab + anti-PD-1, refining the target population and reducing medical risk and cost.

### 2.2 New Preclinical Advances

ICIs show efficacy in many solid tumors but face challenges in glioma. Core challenges include the BBB limiting brain delivery, highly immunosuppressive TME, heterogeneity, scarce tumor-infiltrating lymphocytes (TILs), and dominant TAMs 11. Recent research focuses on novel delivery systems, combination therapies, TME remodeling, and biomarker identification/patient stratification to improve ICI efficacy.

#### 2.2.1 Novel Delivery Strategies

The BBB is a major obstacle that limits the access of ICIs to GBM tumor sites 5. To overcome this, researchers utilized low-intensity pulsed ultrasound combined with intravenous microbubbles to achieve transient, reversible, and localized opening of the BBB, thereby enhancing the concentration of aPD-1 in the sonicated regions 76. Additionally, various delivery systems have been developed, including extracellular vesicles (EV) 77, redox-responsive micelles 78, lipid nanocarriers 79, and nanovesicles 61. These systems successfully crossed the BBB, achieving efficient brain delivery and tumor-specific accumulation of ICIs, improving treatment safety 61, 78, 79. However, these delivery systems are still in the research phase and face translational challenges. For instance, although extracellular vesicles possess good biocompatibility and hold promise as therapeutic carriers, they remain limited by heterogeneity, low yields, inefficient drug loading, and rapid clearance by the mononuclear phagocyte system 80. No EV-based therapy has yet received regulatory approval. Redox-responsive micelles face complex preparation and clinical translation challenges, including amphiphile amount 81, ligand density 82, linkage type 83, polymerization degree 84, reaction conditions 85, and branching degree. These factors collectively influence micelle formation, stability, and scalability. The clinical translation of nanocarriers is hindered by complex physiological barriers, off-target effects of targeting ligands, inherent issues of stability and toxicity, as well as challenges in scalable manufacturing and batch-to-batch consistency 86.

#### 2.2.2 Combination Therapy Strategies

Single ICI efficacy is limited 71. Researchers explored combinations with chemotherapy 79, radiotherapy 87, photodynamic therapy 88, CAR-NK therapy 89, etc., showing synergy. Moreover, dual/multiple immune checkpoint blockade (ICB) (e.g., PD-1/CTLA-4 87, αVβ8/PD-1 90) enhances immune responses 87. Furthermore, combining ICIs with epigenetic regulators such as Zinc Finger Protein 638 (ZNF638) inhibition 91 and CpG-STAT3ASO 81 has demonstrated potential to restore immune sensitivity in preclinical settings 91.

#### 2.2.3 Tumor Microenvironment Remodeling

The GBM TME is highly immunosuppressive, dominated by TAMs and MDSCs 61. Improving this state is key to enhancing ICI efficacy. Strategies include 1) TAM reprogramming (M2 to M1) via inhibiting difluoromethylornithine (DFMO) 61 or Actin Related Protein 2/3 Complex Subunit 1B (ARPC1B) 92; 2) inhibiting immunosuppressive molecules (Nitric Oxide (NO), ROS, IL-10) to protect T cell function 93; 3) modulating B cell function to overcome Transforming Growth Factor β (TGFβ)-mediated immunosuppression 90.

#### 2.2.4 Biomarker Identification

Identifying predictive biomarkers is crucial for patient stratification and personalized therapy. Potential biomarkers explored include 1) molecular markers (PD-L1, ZNF638, ARPC1B) 91, 92; 2) cellular markers (TAMs, B cells, NKT cells) 89, 90, 92; 3) immune microenvironment features (double-stranded RNA (dsRNA), IFN signaling) 91; 4) spatial transcriptomics revealing cell interaction networks 90.

## 3. Adoptive Immune Cell Therapy

Adoptive immune cell therapy is a treatment method in which immune cells with anti-tumor activity are screened, expanded, activated, or genetically engineered *in vitro*, and then infused back into the patient's body to help the patient's immune system recognize and attack cancer cells 94. During the 1980s to 2000s, lymphokine-activated killer (LAK) cells and tumor-infiltrating lymphocytes (TILs) were explored clinically for glioma treatment 95. Early studies by Jacobs et al. demonstrated the feasibility of intratumoral LAK cell injection 96; however, clinical efficacy was limited and responses were transient, with subsequent trials confirming these therapeutic constraints 97, 98. TIL-based approaches faced challenges including low TIL abundance in glioma and functional exhaustion within the immunosuppressive TME 99. However, a recent study using IL-2/IL-15/IL-21-expanded TILs reported complete tumor regression in a glioblastoma patient, suggesting renewed potential for this strategy 100. Around 2000s-mid 2010s, engineered T cells emerged. CAR-T therapy genetically modifies patient T cells to express CARs targeting specific TAAs, enabling tumor cell recognition and killing 101. T cell receptor-engineered T cells (TCR-T) differ by introducing optimized TCR genes to recognize intracellular antigens presented by major histocompatibility complex (MHC) molecules 102. From late 2010s-present, new generation therapies arose, including multi-target CAR-T, logic-gated CAR, armored CAR-T, CAR-NK, CAR-M, neoantigen-targeting CAR-T/TCR-T, combination therapies, and gene-edited universal products 103.

According to the data collected, most of the literature considers adoptive cell therapy to have a good safety profile, with the most serious adverse event usually grade 3 fever 104, 105. However, recent literature has reported that this therapy is associated with the occurrence of cognitive impairment in patients, suggesting that its safety needs to be further reviewed. In glioma patients specifically, neurological events occur in approximately 41% of cases across early-phase trials 106. Mechanistic studies in mouse models have demonstrated that CAR-T therapy can disrupt hippocampal neurogenesis and oligodendroglial homeostasis, leading to cognitive deficits 107, and similar reactive microglial states have been confirmed in human brain tissue from patients who received CAR-T therapy for brain tumors 107. Furthermore, authoritative guidelines from the EBMT and reviews in Nature Reviews Neurology have identified movement and neurocognitive toxicity as emerging concerns across both hematologic and solid malignancies, including CNS tumors 108, 109. These converging lines of evidence underscore the need for further scrutiny of the long-term safety of adoptive cell therapy.

### 3.1 Clinical Trials

Since 2015, CAR-T cell therapies for glioma have entered clinical trials. Targets in CAR-T trials primarily include 1) TAAs: EGFRvIII 13, 14, 110; 2) Interleukin-13 Receptor subunit alpha-2 (IL13Rα2) 111, 112; 3) Disialoganglioside GD2 (GD2) 113; 4) Matrix Metalloproteinase-2 (MMP-2) 114.

#### 3.1.1 EGFRvIII Target

EGFRvIII results from an in-frame deletion of exons 2-7 and mutation at the exon 1/8 junction 14. It is a tumor-specific, oncogenic, immunogenic epitope 14. Expressed in ~25-30% of nGBM cases, its expression is spatiotemporally heterogeneous, can re-emerge, and varies greatly within a tumor 14. The efficacy of anti-EGFRvIII CAR-T cell therapy alone has proven to be limited 14. After treatment, the upregulation of inhibitory molecules, especially PD-L1, in the TME is accompanied by increased infiltration of Tregs, suggesting that the combination of ICIs may improve the efficacy 14. However, a Phase I trial of repeated anti-EGFRvIII CAR-T infusions + Pembrolizumab was ineffective 13. While safe and tolerable, disease control was limited, with most patients progressing within months 13. Reasons included limited peripheral CAR-T cell expansion/persistence, difficulty infiltrating the tumor, EGFRvIII heterogeneity/antigen loss, and increased Tregs/TAMs post-treatment, indicating Pembrolizumab alone couldn't reverse the highly suppressive TME 13. A Phase I trial of bivalent CAR-T cells targeting EGFR epitope 806 and IL-13Rα2 via intracerebroventricular delivery for EGFR-amplified rGBM showed safety and feasibility 110. 62% had tumor shrinkage, a few had durable control 110. The trial enrolled EGFR-amplified patients but not based on IL-13Rα2 expression, potentially affecting efficacy 110. In five re-infused patients, CAR-T expansion/persistence was worse than the first infusion, with short PFS, suggesting immune clearance or resistance mechanisms 110.

#### 3.1.2 IL13Rα2 Target

**Figure 3:**
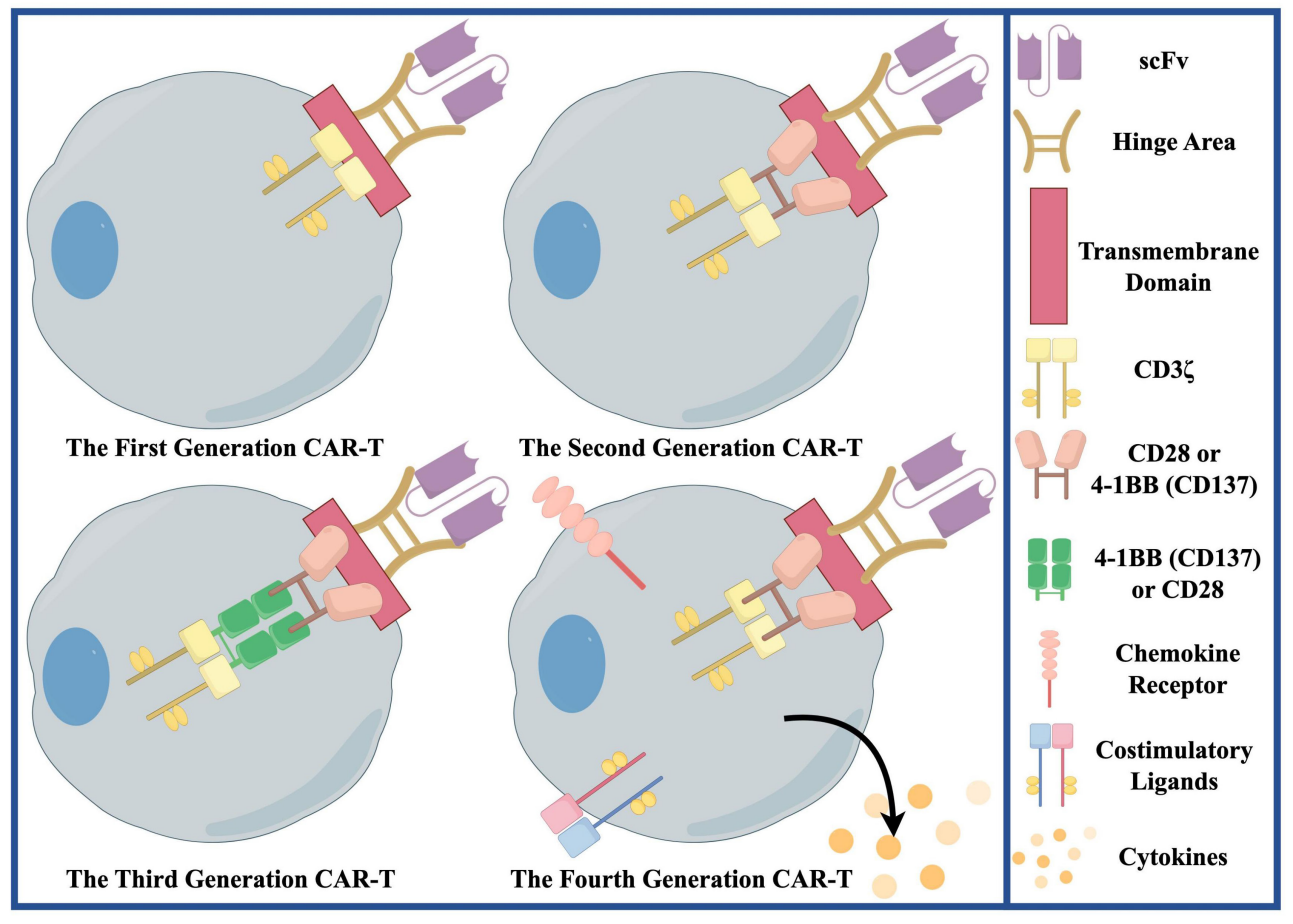
This figure shows the engineered structure of the first to fourth-generation CAR-T. The core structure of first-generation CAR-T is an extracellular single-chain variable fragment (scFv) plus a single intracellular signaling domain (usually CD3ζ). The core structure of the second-generation CAR-T is extracellular scFv + a costimulatory signal domain + CD3ζ. The core structure of the third-generation CAR-T is extracellular scFv + two or more costimulatory domains + CD3ζ. The core structure of the fourth-generation CAR-T integrates additional genetic elements on the basis of the second-generation CAR, so that it can secrete specific cytokines or express additional receptors. The structures of the fourth-generation CAR-T are diverse, and only the cytokine secreting fourth-generation CAR-T is shown in the figure.

IL13Rα2 is a high-affinity IL-13 decoy receptor, negatively regulating IL-13 signaling 111. It is expressed in ≥ 75% of GBM patients, overexpressed in > 50%, correlating with shorter survival 111. It is expressed on GSCs, differentiated tumor cells, and TAMs/MDSCs, but not significantly in normal brain 111. Its expression correlates with the mesenchymal subtype 111. This first-in-human trial evaluated an off-the-shelf, steroid-resistant allogeneic CAR-T therapy targeting IL13Rα2 in rGBM 112. By using healthy donor T cells, it overcomes the manufacturing time, cost, and feasibility limitations of autologous CAR-T 112. This therapy uses zinc-finger nucleases to knock out the glucocorticoid-receptor gene in T cells so that they remain functional in the presence of dexamethasone 112. In terms of clinical outcomes, treatment was well tolerated, with local tumor necrosis or shrinkage in most patients 112. However, antitumor activity was transient, and all patients eventually experienced disease progression 112. The lack of durability of CAR-T cells is a major factor limiting efficacy, in part because of the use of first-generation CAR constructs **(Figure 3)** containing only the CD3ζ signaling domain, which lack the costimulatory domain and result in limited expansion and survival 112. In addition, because HLA matching was not performed, one of the six patients developed anti-CAR antibodies, suggesting the presence of host immune rejection 112. Future improvements include the use of second-generation or higher-generation CAR designs with costimulatory domains, optimized gene editing (e.g., knockout of the TCR to avoid graft-versus-host disease), and the exploration of manufacturing processes that preserve the stem-like properties of T cells and prolong their survival 112.

#### 3.1.3 GD2 Target

GD2 is a disialoganglioside, and its expression is highly specific 113. In normal tissues, it is mainly distributed in the neuronal cell membrane 113. However, it is highly expressed in a variety of malignant tumors 113. In GBM, GD2 is enriched on tumor cells and specifically overexpressed on GSCs. ~80% of diffuse intrinsic pontine gliomas express GD2 113. A trial of GD2-specific 4SCAR-T cells for GD2-positive GBM used a fourth-generation CAR design with an inducible suicide gene 113. The results showed that both intravenous and intracavitary injections were safe and tolerable 113. Median survival from infusion was 10 months 113. Regarding CAR-T cell kinetics, they expanded in peripheral blood for 1-3 weeks, then persisted at low frequency 113. Importantly, post-infusion tumor resection showed GD2 antigen loss (mechanism unknown), suggesting need for multi-target therapy 113. Additionally, after treatment, TAMs increased and tumor-infiltrating NK cells decreased, suggesting that the immunosuppressive microenvironment still needs to be improved 113.

#### 3.1.4 MMP-2 Target

Metalloproteinase MMP-2 is widely expressed in GBM cells, and its main role is to degrade collagen Ⅳ, the main component of the basement membrane of the extracellular matrix 114. In gliomas, overexpression of MMP-2 is closely related to the malignant behavior of tumors 114. It accelerates the malignant progression of glioma by destroying the BBB, inhibiting immune cell infiltration, and mediating immune evasion 114. The CLTX-CAR T-cell study is the first to exploit the ability of CLTX, a peptide derived from scorpion venom, to treat patients with recurrent, MMP-2-expressing GBM through the mechanism of broad MMP-2 binding to glioma cells 114. Pharmacokinetically, CAR T cells were persistently detected in tumor cavity fluid (TCF) and at low levels peripherally, proving intratumoral delivery allows transport 114. In terms of safety and efficacy, intratumoral infusion was tolerable with manageable adverse events (AEs), achieving a disease control rate of 75%, although the small sample size precluded assessment of survival benefit 114. However, several limitations were identified. MMP-2 expression on tumor vasculature, fibroblasts, and myeloid cells poses a potential risk 114. Furthermore, the inhibitory TME may limit efficacy, as stromal and myeloid components may drive T cell exhaustion, suggesting that combination strategies targeting the TME could improve outcomes 114. Future directions include modifying peptide ligand/CAR structure for better targeting, enhancing T cell persistence/proliferation, and combining therapies 114.

### 3.2 New Preclinical Advances

CAR-T therapy has demonstrated significant clinical efficacy in hematologic malignancies, but its application in glioma remains limited. Major challenges: 1) High antigen heterogeneity; 2) Immunosuppressive TME factors (TGF-β, PD-L1); 3) CAR-T cell exhaustion from chronic antigen exposure; 4) BBB limiting migration; 5) Antigen loss/downregulation post-treatment 94. Recent innovations to overcome these include: 1) Universal targeting platforms (e.g., monomeric streptavidin 2-based Chimeric Antigen Receptor T cells (mSA2 CAR-T)) 115; 2) Dual/multi-target CAR designs 116; 3) Armored CAR-T cells 116; 4) Sonogenetically controlled CAR-T 117; 5) Combination therapies (e.g., with oncolytic viruses, small molecules) 118, 119; 6) New targets (e.g., Glycoprotein A Repetitions Predominant, Human Leukocyte Antigen-E) 120, 121. Conversely, several other CAR-T strategies have demonstrated limited efficacy or encountered significant challenges in preclinical glioma models. These include permanently expressed CAR constructs associated with off-tumor toxicity 122, and B7-H3-targeted CAR-T cells that have yet to overcome intratumoral heterogeneity 123. Promising preclinical studies are listed below.

#### 3.2.1 Universal CAR-T Platform: mSA2 System

The mSA2 CAR-T platform is a revolutionary universal strategy 115. It uses mSA2 as the CAR's antigen-binding domain, mediated by biotinylated antibodies 115. Its advantage is that the targeting specificity of CAR-T cells can be rapidly changed by simple replacement of biotinylated antibodies, without redesign and manufacture of new CAR constructs, so that rapid target switching, multi-target combination, overcoming antigen escape, and reducing manufacturing costs can be achieved 115. Researchers developed biotinylated antibodies for GBM markers CD276 (B7-H3), EPHA2, CD70, IL13Rα2 115. *In vitro*, mSA2 CAR-T cells specifically recognized primary GBM cell lines antigen-dependently, simultaneously targeting heterogeneous subpopulations, clearing GBM cells within 20 hours via live imaging 115. In immunodeficient mouse orthotopic GBM models, intracranial injection of mSA2 CAR-T pre-loaded with anti-CD70 or anti-CD276 antibodies significantly suppressed tumor growth, induced apoptosis, and extended survival 115. This therapy provides a good safety foundation for clinical translation. In a mouse model of GBM, mSA2 CAR-T cells did not interact with brain endogenous biotin, thereby reducing the risk of off-target cross-reactivity 115.

#### 3.2.2 Checkpoint Antibody Receptor-modified (ARMed) CAR-T Cells

The BBB blocks antibody entry into the CNS, a major reason for limited efficacy in anti-PD-1 + CAR-T trials 94. To overcome this, researchers developed fourth-generation ARMed CAR-T, combining EGFRvIII-targeting CAR with anti-PD-1 minibody secretion 118. The advantage of this therapy is that ARMed CAR-T is able to cross the BBB and secrete microantibodies at the tumor site, overcoming the BBB limitation of systemic delivery 118. Mice treated with EGFRvIII ARMed CAR-T had significantly smaller brain tumors versus controls, with a median OS of 34 days and 10% surviving to day 50 (study end) 118. Additionally, no dose-limiting toxicities were observed, and human T cells remained detectable in peripheral blood at day 29 post-infusion, comparable to CAR T cells alone 118.

#### 3.2.3 NR4A3 Deficiency + FOS Overexpression Synergy Overcomes CAR-T Exhaustion

NR4A family genes (NR4A1, NR4A2, NR4A3) are upregulated in TILs, especially terminally exhausted T cells 124. FOS expression is highest in CD8⁺ naive populations and is co-expressed with stemness genes including TCF7, CCR7, IL7R, and LEF1 124. Based on this, researchers knocked down NR4A3 and overexpressed FOS (FOS OE/NR4A3 KD) in CAR-T cells 124. Modified CAR-T cells showed long-term tumor suppression, significantly extended mouse survival, and widespread tumor infiltration 124. This provides a novel genetic modification strategy for solid tumor CAR-T, enhancing stemness and anti-exhaustion capacity by targeting NR4A3 and FOS, offering a preclinical basis for delaying progression 124.

#### 3.2.4 CD44/CD133 Dual-Target IL7Rα Armored CAR-T Cells

IL7Rα is key for T cell survival/proliferation 116. Armoring CAR-T with ΔIL7Rα intracellular domain enhances tumor-killing 116. Targeting both CD44 and CD133, classic GSC biomarkers, aims to clear GSCs comprehensively, preventing antigen escape 116. Since CD44 is also on T cells, to prevent CAR-T fratricide, researchers modified the CAR structure; Tanζ-T28 became the ideal base, successfully constructing Tanζ-T28 CAR-T cells 116. Evaluating Tanζ-T28 CAR-T in glioblastoma organoid (GBO) models showed decreasing GBO volume, successful CAR-T infiltration, memory T cell enrichment in brain tissue, reduced checkpoint marker expression, and upregulated effector function genes 116. In addition, a clinical trial (NCT05577091) is ongoing 116.

In recent years, with the continuous advancement of innovative research on CAR-T therapy, the field has encountered challenges in clinical translation. Overall, the clinical translation of CAR-T therapy is hindered by high costs, complex regulatory compliance, substantial quality variability in manufacturing processes, and a widespread shortage of infrastructure and specialized personnel 125. However, universal platforms such as mSA2 CAR-T may help reduce costs and accelerate the clinical translation process 115.

## 4. Oncolytic Virus Therapy

**Figure 4:**
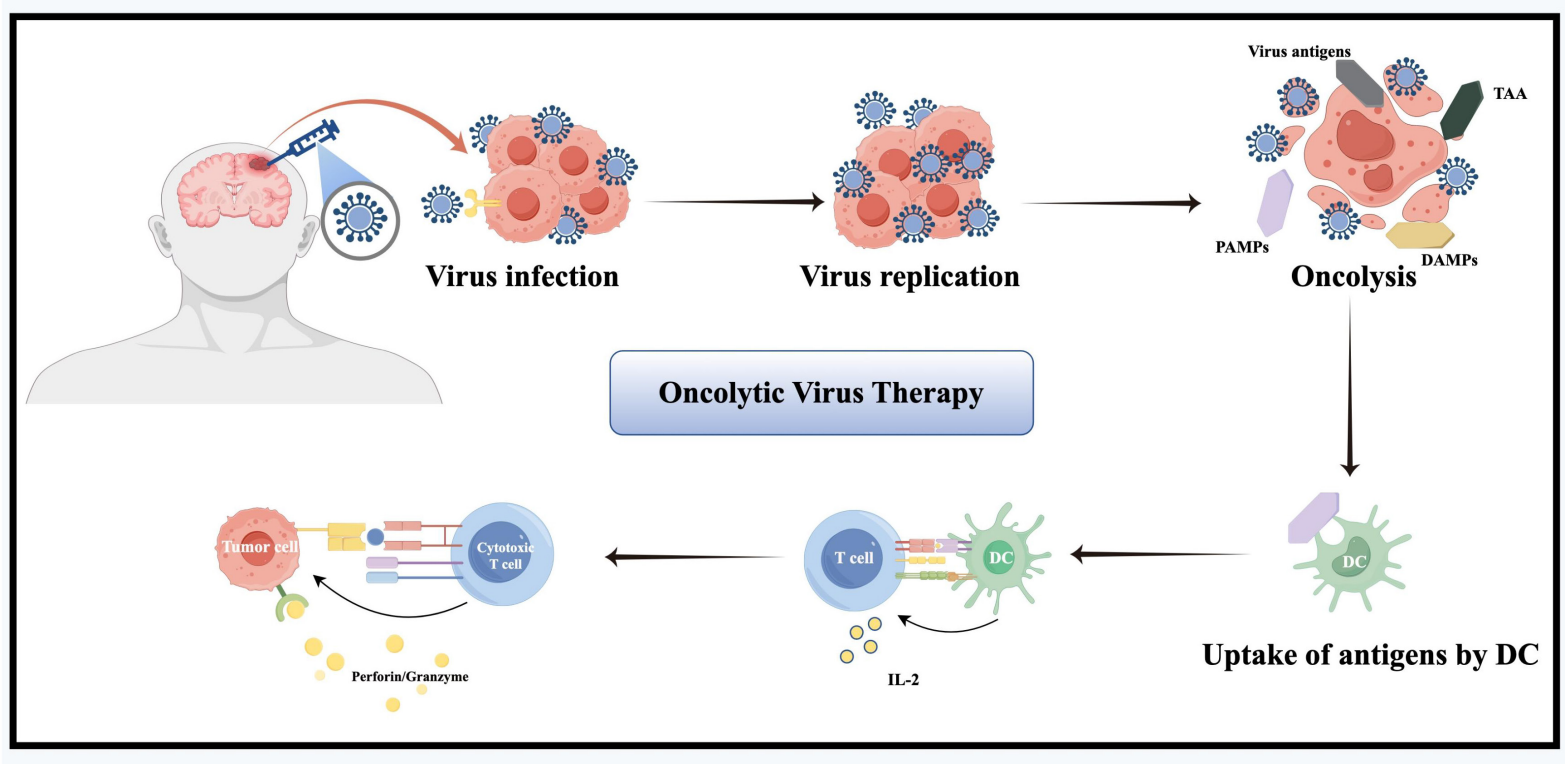
The figure illustrates the oncolytic effect mediated by oncolytic viruses following intratumoral injection. This effect promotes the release of a large amount of antigens from the tumor, including tumor-associated antigens (TAAs), damage-associated molecular patterns (DAMPs), pathogen-associated molecular patterns (PAMPs), and viral antigens, thereby facilitating the activation of immune cells and their capacity to kill tumor cells.

Oncolytic virus therapy uses genetically engineered, weakly pathogenic viruses to selectively induce ICD in cancer and stromal cells 7 **(Figure 4)**. It works via multiple mechanisms: direct oncolysis, immune activation, TME remodeling, and vascular disruption 126. In the early 21st century, clinical trials initially verified the safety and feasibility of herpes simplex virus type 1 (HSV-1) G207 127, reovirus adenovirus 128, NDV-HUJ oncolytic virus 129 and other modified viruses. A Phase I trial of G207 combined with radiotherapy demonstrated acceptable safety and radiographic responses in rGBM patients, with six of nine patients achieving stable disease or partial response 127. By 2010, the safety and feasibility of DNX-2401 (Delta-24-RGD) and recombinant poliovirus (PVSRIPO) were established 130, 131. A pivotal Phase I study of intratumoral DNX-2401 in rGBM reported a 20% 3-year survival rate, including one patient who achieved complete remission lasting three years 130. Notably, post-treatment tumor analysis revealed CD8^+^ T-cell infiltration, providing clinical evidence of "cold"-to-"hot" tumor conversion 130. Concurrently, a Phase I trial of PVSRIPO in rGBM demonstrated a 21% 3-year survival rate versus 4% in historical controls, highlighting its potential to induce durable antitumor immunity 131. Since the late 2010s, emerging strategies have focused on combination approaches with ICIs, chemoradiotherapy, and the design of next-generation oncolytic viruses 126, 132-135.

### 4.1 Clinical Trials

Oncolytic virus platforms in trials over the past decade include: 1) measles virus 136; 2) adenovirus 132, 137, 138; 3) herpes virus 139, 140.

#### 4.1.1 Oncolytic Measles Virus

Measles virus is a single-stranded, negative-sense RNA virus. It naturally targets cells via receptors (CD46, Nectin-4), which are often overexpressed on cancer cells 136. Harnessing this property, oncolytic measles viruses are engineered for enhanced efficacy/safety, overcoming natural limits, and allowing replication monitoring 136. A prime example is the Measles Virus-based Carcinoembryonic Antigen-expressing Oncolytic Virus (MV-CEA), derived from the safe Edmonston vaccine strain 136. It causes minimal normal tissue damage, uses CD46 for selective infection, has a strong bystander effect, and expresses soluble CEA for monitoring replication 136. In clinical testing, this therapy was well tolerated and safe, with a median OS of 11.6 months and a 12-month OS rate of 45.5% in patients with refractory rGBM, which was superior to historical controls 136. Notably, pre-existing immunity did not prevent virus replication within the tumor 136. Instead, tumor-intrinsic interferon-stimulated gene (ISG) levels emerged as the key determinant of viral replication 136. Specifically, high ISG signaling inhibited viral replication, thereby limiting therapeutic benefit 136. Therefore, this therapy may be best suited for low ISG patients; for high ISG, combining with JAK and STAT inhibitors could block interferon response and enhance replication 136. Additionally, CD8^+^ T cell infiltration observed post-treatment waned in subsequent samples from some patients, suggesting single/limited treatments may not sustain long-term immune activation 136. Future directions include developing measles strains expressing H. pylori neutrophil-activating protein for enhanced immunostimulation or combining measles virus with ICIs 136.

#### 4.1.2 Oncolytic Adenovirus

Adenovirus is a non-enveloped dsDNA virus. Genetic modification enables tumor-selective replication 141. Several adenoviral platforms have been clinically assessed for GBM, including Ad-TD-nsIL12 137, DNX-2401 132, and NSC-CRAd-S-pk7 138. Among these, Ad-TD-nsIL12 is a third-generation adenovirus with multiple genetic modifications 137. Specifically, deletion of E1ACR2 and E3gp19K enabled tumor-specific replication, whereas deletion of E1B19K enhanced antitumor immunity, whereas E1B55K and E3B overcame previous limitations of impaired viral replication capacity and M2-type macrophage infiltration 137. Furthermore, the IL-12 signal peptide was modified to create a non-secreting form (nsIL12), enabling sustained, low-level IL-12 expression within the TME while avoiding systemic IL-12 toxicity such as lethal inflammatory syndrome 137. Clinically, a Phase I trial demonstrated that Ad-TD-nsIL12 was tolerable at 1×10¹⁰ vp, with dose-limiting toxicity occurring at 5×10¹⁰ vp. Most adverse events were grade 1-2 and resolved within 48 hours 137. Median OS was 5.1 months (range: 3.1-21.2 months) 137. Notably, one patient achieved complete response and one achieved partial response; both responders had IDH1 mutation and MGMT expression, suggesting that exploring IDH mutant sensitivity to oncolytic viruses warrants further investigation 137. Mechanistically, late TME analysis revealed dominance by M2 macrophages (CD163⁺), which may promote tumor progression 137. Therefore, combining Ad-TD-nsIL12 with M2 inhibitors or reprogrammers to promote M1 polarization could enhance therapeutic efficacy 137.

Another platform, DNX-2401 (tasadenoturev/Delta-24-RGD), is a conditionally replicating adenovirus featuring a 24-bp deletion in E1A for selective replication and an RGD peptide insertion in the fiber knob to improve GBM cell infectivity via integrin binding 132. In a multicenter Phase I/II trial (NCT02798406), DNX-2401 combined with Pembrolizumab was safe and tolerable 132. Median survival in GBM was 12.5 months, with an objective response rate (ORR) of 10.4%, which was not statistically different from the preset 5% control rate 132. Importantly, post-treatment analysis revealed upregulation of several immune checkpoints (TIGIT, LAG3, B7-H3), suggesting that combining multiple ICIs post-treatment might improve efficacy 132. Furthermore, an exploratory analysis suggested that patients with a TME^medium^ subtype characterized by moderate immune infiltration, medium PD-1 expression, and absence of multiple immunosuppressive factors may be more likely to derive clinical benefit from this approach 132.

#### 4.1.3 Oncolytic Herpes Virus

HSV is a neurotropic dsDNA virus with a large genome (~150kb) 142. Over the past decade, G47Δ has emerged as a key oncolytic HSV-1 variant 139, 140. It is a third-generation, triple-mutated virus constructed by deleting the α47 gene and overlapping the US11 promoter from its parental virus G207, which enhances tumor-specific replication and immunogenicity 139. In clinical studies, previous Phase I/II trials confirmed the safety of G47Δ in progressive GBM, with patients achieving a median survival of 7.3 months (range: 1-14 months) 140. A subsequent Phase II trial for residual or recurrent GBM showed more impressive results: median OS from G47Δ start was 20.2 months, with a 1-year survival rate of 84.2%—far exceeding the 15% historical control 139. Mechanistically, biopsy after treatment revealed a sustained increase in CD4⁺ and CD8⁺ T cells without an increase in Foxp3⁺ Tregs, suggesting that G47Δ can effectively improve the immunosuppressive microenvironment and promote immune infiltration 139. Based on these findings, G47Δ was approved in Japan in June 2021, becoming the country's first oncolytic virus product 139. However, distant new lesions, CSF dissemination, and local recurrence around the target were common, indicating that local therapy struggles to control systemic or diffuse progression 139. Additionally, imaging proved inadequate for accurately evaluating efficacy, underscoring the critical need for novel assessment methods 139.

### 4.2 New Preclinical Advances

Oncolytic virus therapy shows great potential but faces challenges: poor targeting, high toxicity, poor drug control, systemic delivery limited by BBB, weak virus infiltration, host immune clearance, and immunosuppressive TME limiting immune activation. Recent strategies in preclinical research have made important progress. Innovations in virus platforms, delivery strategies, combination therapies, and metabolic regulation are summarized below.

#### 4.2.1 New Oncolytic Virus Platforms

New platforms focus on improving targeting, reducing toxicity, and enabling drug control. 1) Zika virus (ZIKV), 2) infectious bursal disease virus (IBDV), 3) human cytomegalovirus (HCMV), and 4) myxoma virus (MYXV) platforms provide strong support 126, 133, 143-145. 1) ZIKV shows selective targeting of GSCs, with natural neuroprogenitor tropism and SOX2⁺ cell preference 126, 143. 2) IBDV is a dsRNA non-human virus, avoiding pre-existing immunity; it infects tumor and myeloid cells 133. 3) HCMV based on AD169 strain: deleted UL1-UL20/UL/b regions to reduce virulence; restored pentamer complex (gH/gL/pUL128-131) function to enhance tropism; integrated paramyxovirus glycoprotein H/F complex for EGFR targeting; added tet-off system for drug-controlled IL-12 expression 144. 4) MYXV is an enveloped dsDNA poxvirus. A MYXV construct lacking the M011L gene (vMyx-M011L-KO/EGFP) induced apoptosis in infected brain tumor-initiating cells 145.

#### 4.2.2 Innovative Delivery Strategies

Cell carrier-mediated delivery, surface modification for BBB penetration, and combination with photodynamic therapy (PDT) improve virus delivery to tumors 145-148. 1) Macrophage carriers protect virus from neutralizing antibodies, effectively infiltrate glioma tissue, and have strong tumor tropism 146. 2) Adipose-derived stem cell carriers have low immunogenicity (allowing allogeneic use), strong tumor tropism, protect virus from antibodies/T cells, and maintain migration ability post-infection 145. 3) Cholesterol-modified adenovirus (OA@Cho) uses LDL receptor-mediated transcytosis across BBB for precise glioma targeting/delivery 147. 4) PDT-generated ROS damage cancer cells, increase tumor vascular permeability, induce ICD, thereby increasing virus susceptibility, promoting virus entry, and enhancing anti-tumor immunity 148.

#### 4.2.3 Combination Therapy Strategies

Combining oncolytic viruses with chemotherapy, ICIs, metabolic inhibitors, PDT, etc., shows synergistic potential for improved efficacy 133-135. For example, when IBDV is combined with TMZ, IBDV can enhance the cytotoxic activity of TMZ, and TMZ not only does not impair the susceptibility of GBM cells to IBDV, but can enhance the ability of viral replication and promote viral release 133. Triple therapy (a bromodomain-containing protein 9 (BRD9) inhibitor + oHSV + anti-PD-1/CTLA-4) showed potent anti-tumor effect, nearly eradicating tumors; long-term survivors showed protective immunity upon re-challenge 134.

#### 4.2.4 Metabolic Regulation Intervention

Metabolic reprogramming and targeting metabolic pathways can reduce virus-induced lipid peroxidation 135. For example, combining oHSV with an IDH R132H inhibitor restored the sensitivity of IDH-mutant glioma to oHSV, inducing ferroptosis and anti-tumor immunity 135. Mechanistically, this combination blocked glycolysis, cleared ROS, inhibited glutamine metabolism, and suppressed Protein Kinase C in immunocompetent mouse IDH-mutant tumor models, significantly improving virus therapy efficacy 135. However, the use of metabolic reprogramming inhibitors must ensure selective toxicity to tumor cells, thereby preventing metabolic disruption in normal cells that could render them susceptible to oncolytic virus infection 149.

### 4.3 Biomarkers and Patient Stratification

Analysis of GBM trial (NCT03152318) data revealed several predictive biomarkers: 1) Low BRD9 mRNA expression correlated with significantly longer PFS 134. 2) METTL14-low gliomas are more sensitive to oHSV-1 150. 3) Wild-type IDH gliomas are more sensitive to oHSV-1 134. 4) High PD-L1/PD-1 expression suggests potential benefit from combination ICB 135. 5) High glycolysis patients may be sensitive to metabolic reprogramming. In these cases, consider combining glycolysis inhibitors 135. 6) Patients with high oxidative phosphorylation may benefit from oHSV monotherapy 135. 7) Immune "hot" tumors with rich T cell infiltration suit viral immunotherapy and may benefit from ICIs 134. 8) Immune "cold" tumors with scarce T cell infiltration (especially GBM) need virus-induced immune activation. For such patients, consider higher doses, multiple administrations, or combining immunomodulators 134.

In recent years, advancements in oncolytic virotherapy have provided new strategies for the treatment of glioma. However, the translation of these therapies from research to clinical application remains hindered by multiple challenges, including safety risks and heterogeneity associated with animal-derived components, difficulties in maintaining viral activity and stability, and the urgent need to upgrade purification technologies toward large-scale, automated, and intelligent manufacturing 151.

## 5. Common Challenges Shared across Different Immunotherapies

Although the immunotherapeutic strategies described above have shown promise in preclinical models, they have largely failed to translate into durable survival benefit in clinical trials **(Table 1)**. This suggests common barriers across different therapeutic modalities. This section systematically analyzes the core mechanisms underlying these failures, including 1) immunosuppression in the TME 152, 153, 2) tumor antigen heterogeneity and escape 13, 154, 3) blood-brain barrier limitations for drug delivery 155, 156, and 4) adaptive resistance under therapeutic pressure 153, 157.

### 5.1 Immunosuppression in the Tumor Microenvironment

**Figure 5:**
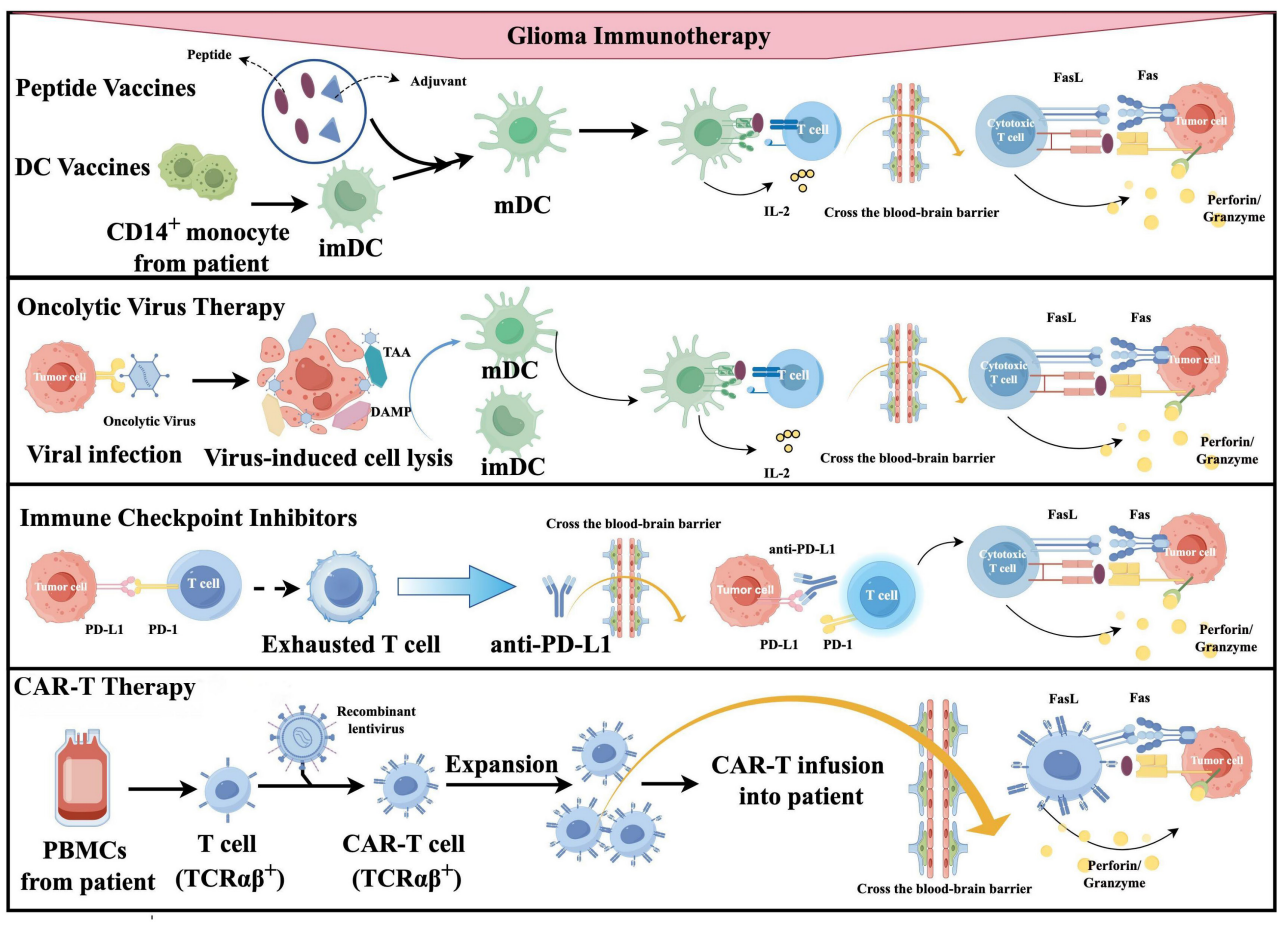
Schematic overview of four major immunotherapeutic strategies for glioma. 1) Peptide vaccines and dendritic cell (DC) vaccines: Peptide vaccines deliver tumor-associated antigens (TAAs) in combination with adjuvants, thereby inducing DC maturation *in vivo*. DC vaccines are generated by differentiating patient-derived CD14⁺ monocytes into immature DCs, followed by loading with tumor antigens. Mature DCs promote the activation and differentiation of T cells. Activated T cells cross the blood-brain barrier (BBB) and exert anti-glioma effects through Fas/FasL-mediated apoptosis and the perforin/granzyme release pathway. 2) Oncolytic virus therapy: Oncolytic viruses selectively infect and lyse glioma cells, releasing TAAs and damage-associated molecular patterns (DAMPs), which in turn activate DCs and initiate T cell responses, ultimately leading to tumor cell killing. 3) Immune checkpoint inhibitors: These inhibitors block immune checkpoint signaling pathways, reverse T cell exhaustion, and thereby eliminate glioma cells. 4) Chimeric antigen receptor T (CAR-T) cell therapy: Patient-derived peripheral blood mononuclear cells (PBMCs) are isolated, genetically modified to express chimeric antigen receptors (CARs), expanded *ex vivo*, and then infused back into the patient. CAR-T cells cross the BBB and specifically target and kill glioma cells.

Regardless of the immunotherapeutic strategy employed, its ultimate antitumor efficacy depends on the effective activation of a functional immune system 158 **(Figure 5)**. However, the TME of glioma, particularly glioblastoma, is characterized by profound immunosuppression, rendering immune cells—whether vaccine-induced T cells 159 or CAR-T cells 13, 14—prone to inactivation or exhaustion upon entry into the tumor site.

#### 5.1.1 Impact on Vaccines

Vaccines require the presentation of antigens to DCs to subsequently activate T cells 160. However, in the immunosuppressive TME, dendritic cell function is impaired, and activated T cells are also unable to effectively infiltrate and proliferate 160.

#### 5.1.2 Impact on ICIs

The PD-1/PD-L1 immune checkpoint pathway is just one of many immunosuppressive mechanisms 161. The TME also contains abundant Tregs, MDSCs, and inhibitory cytokines such as TGF-β and IL-10 161. Even upon PD-1/PD-L1 blockade, these additional mechanisms continue to effectively suppress T cell function 162.

#### 5.1.3 Impact on CAR-T Cells

Even if CAR-T cells successfully reach the tumor site, they encounter the same immunosuppressive microenvironment, leading to poor persistence, rapid exhaustion, and an inability to sustain long-term antitumor effects 103.

#### 5.1.4 Impact on Oncolytic Viruses

The primary mechanism underlying the antitumor effect of oncolytic viruses is a T cell-mediated, tumor-specific immune response 163. However, the TME can inhibit T cell function through various mechanisms, thereby compromising the efficacy of this therapeutic approach 163.

### 5.2 Tumor Antigen Heterogeneity and Escape

Glioblastoma exhibits significant spatial and interpatient heterogeneity, which is a key factor contributing to immune evasion 164. The expression of recognizable antigens on tumor cells is a prerequisite for vaccine-induced immune responses and for ICIs to relieve T cell suppression 165. For oncolytic viruses, however, direct oncolysis depends on the expression of surface receptors on target cells, yet it does not require that all tumor cells express such receptors 166. As long as a sufficient number of cells express the receptors and become infected, releasing ample damage-associated molecular patterns and tumor-associated antigens, an immune response can be activated to eliminate tumor cells that do not express the receptors 167.

Strategies such as multi-target vaccines 19, dual-target CAR-T cells 116, and "plug-and-play" CAR-T platforms (e.g., mSA2 CAR-T) 115 hold promise for overcoming immune evasion driven by tumor heterogeneity by enabling simultaneous targeting of multiple tumor-associated antigens or flexibly adapting to changes in antigen expression, positioning them as a key direction for future research.

### 5.3 Blood-Brain Barrier Constraints

Glioblastoma is located within the central nervous system, where the unique BBB not only acts as a physical obstacle to drug delivery but also restricts the entry of immune cells from the periphery into the tumor site 168.

#### 5.3.1 Impact on Macromolecular Therapeutics and Oncolytic Viruses

Monoclonal antibodies (such as PD-1/PD-L1 antibodies), CAR-T cells, and oncolytic viruses are all macromolecules or nanoscale particles that are largely incapable of crossing an intact BBB under normal conditions 145-148. Even though tumor growth can disrupt the local barrier, such disruption is heterogeneous and insufficient, resulting in suboptimal drug accumulation 156.

#### 5.3.2 The Impact on Vaccines

Vaccines activate T cells in peripheral lymph nodes, and these activated T cells must successfully cross the BBB to exert their antitumor effects 169. The presence of the BBB significantly limits the infiltration efficiency of these T cells 169. Various delivery strategies, such as nanocarriers 61, 79 and focused ultrasound 76, offer potential approaches to overcome this physical barrier.

### 5.4 Adaptive Resistance and Tumor Evolution

Most studies suggest that tumors undergo dynamic evolution under therapeutic pressure. Even when an initial treatment is effective, tumors can acquire adaptive resistance through multiple mechanisms, including upregulation of alternative immune checkpoint molecules, metabolic reprogramming, or activation of bypass growth signaling pathways 68, 132. However, a few studies have also demonstrated that tumors can evolve even in the absence of pharmacological intervention, under natural conditions 31.

For instance, following treatment with PD-L1 inhibitors, tumors may upregulate the expression of other checkpoint molecules (such as LAG-3, TIM-3, TIGIT, and CTLA-4), thereby achieving "bypass escape" 170. For CAR-T therapy, in addition to direct antigen loss, tumors can also evade immune recognition by downregulating antigen processing and presentation machinery components, such as MHC class I molecules, thereby impairing the antitumor response of endogenous T cells and indirectly promoting immune evasion 171. For vaccines, under the immune pressure induced by treatment, tumor cells may escape immune recognition by directly downregulating target antigen expression or by downregulating antigen processing and presentation machinery components, such as MHC class I molecules, thereby hindering the killing of tumor cells by CD8^+^ T cells 172. Oncolytic virus infection of tumor cells depends on specific cell surface receptors 173. Under therapeutic pressure, tumor cells can resist viral infection by downregulating or losing these receptors 173.

This mechanism reveals the underlying reasons for the transient efficacy of monotherapy and highlights the importance of combination therapy. The advantages of combination therapy can be summarized as follows: 1) Broader target coverage. Whether through the combination of ICIs 70, multi-target vaccines 19, or universal CAR-T platforms 115, the range of target coverage exceeds that of monotherapy, enabling more effective elimination of heterogeneous tumor cell populations. 2) Broader coverage of mechanisms of action. Under therapeutic pressure, tumors can acquire adaptive resistance through multiple mechanisms 68, 132. To circumvent this, combining therapies with distinct mechanisms—such as ICIs with epigenetic modulators 91 or with oncolytic viruses 134—makes it more difficult for tumors to escape. 3) Synergistic effects. The combination of certain therapies, such as chemotherapy with immunotherapy, can produce synergistic effects that achieve "1+1 > 2" therapeutic outcomes, surpassing the simple additive effects of individual agents 39. 4) Improved safety. In a combination therapy setting, the dosage of each therapeutic agent can be reduced accordingly, thereby alleviating target organ toxicity and enhancing the safety and tolerability of treatment 174. In line with the growing recognition of combination strategies, several ongoing clinical trials are evaluating the efficacy of immunotherapy combined with other modalities** (Table 2)**.

In summary, glioma immunotherapy faces a series of interrelated and overlapping challenges, including a complex immunosuppressive TME, high tumor heterogeneity, the restrictive nature of the BBB, and tumor adaptive resistance and evolution. In the future, strategies such as multi-target approaches, optimization of delivery systems, remodeling of the TME, and the development of rational combination therapies hold promise for overcoming these obstacles one by one, ultimately achieving breakthroughs in glioma immunotherapy.

## 6. Biomarkers: From Classification to Combination Strategies

The biomarkers discussed in this manuscript have distinct functional roles in clinical practice. To establish a unified classification framework, this section categorizes them into three groups: predictive biomarkers, prognostic biomarkers, and biomarkers guiding combination therapy selection.

### 6.1 Predictive Biomarkers

Predictive biomarkers are defined as biological characteristics that can be objectively measured and indicate the likelihood of response to a specific therapeutic intervention 175. These markers are critical for patient selection in targeted therapy and immunotherapy, enabling the identification of patients most likely to derive clinical benefit while minimizing unnecessary toxicity 175. **Table 3** summarizes key predictive biomarkers discussed in this review, along with their associated therapies and current evidence.

### 6.2 Prognostic Biomarkers

#### 6.2.1 IDH-wildtype glioblastoma

MGMT promoter methylation is one of the most well-established prognostic biomarkers in IDH-wildtype glioblastoma, with its methylation status associated with significant improvements in OS and PFS 176, and it retains prognostic value even in the context of adjunctive therapies such as tumor-treating fields 177. TERT promoter mutations occur in approximately 80% of IDH-wildtype glioblastomas 178; however, their prognostic significance remains controversial. Some studies have associated TERT promoter mutations with poorer survival 179, whereas others suggest a synergistic interaction between TERT promoter mutations and MGMT methylation status, with patients harboring both alterations exhibiting the longest survival 180. In addition, chromosome 7 gain and chromosome 10 loss, along with EGFR amplification, are characteristic genomic alterations in IDH-wildtype glioblastoma 181 and are closely associated with worse OS and PFS 182. PTEN mutations, observed in approximately 40% of cases, are likewise associated with poor prognosis and treatment resistance 183.

#### 6.2.2 IDH-mutant astrocytomas

In IDH-mutant diffuse astrocytomas, tumor grade is a key prognostic factor, with grade 3 tumors exhibiting a median OS of 8.1 years, whereas grade 4 tumors show a significantly shorter median OS of 4.7 years (p < 0.05) 184. Homozygous CDKN2A/B deletion is strongly associated with worse survival outcomes; patients with this deletion have a median OS of only 1.8 years compared with 5.5 years in those without the deletion (p < 0.001) 2. The presence of IDH mutation has now been established as a favorable prognostic factor in diffuse astrocytomas and oligodendrogliomas 185. MGMT promoter methylation is more prevalent in IDH-mutant gliomas and is independently associated with improved OS and PFS when treated with TMZ; however, its predictive value appears to be largely limited to grade 4 tumors 186. In addition, TP53 mutations and ATRX loss frequently co-occur, indicating genomic instability, and are generally associated with favorable prognosis 187; patients with ATRX mutations are younger and have longer OS 188.

#### 6.2.3 IDH-mutant and 1p/19q-codeleted oligodendroglioma

Oligodendrogliomas have long been recognized as having a favorable prognosis, with their defining feature being the concurrent presence of IDH1/2 mutations and 1p/19q codeletion 2. The molecular landscape of oligodendrogliomas includes frequent TERT promoter mutations 189, retained ATRX expression, and absence of p53 accumulation, which is consistent with the mutual exclusivity between 1p/19q codeletion and TP53 or ATRX alterations 190. In a subset of cases, CDKN2A/B deletions may be detected, an alteration associated with more aggressive tumor behavior and poorer prognosis 191.

### 6.3 Biomarkers for Combination Therapy Selection

The rational design of combination therapies increasingly relies on biomarkers that identify synergistic vulnerabilities.** Table 4** outlines representative biomarkers and their associated combination approaches.

## 7. Conclusions

Recent progress in glioma immunotherapy has been substantial, particularly in efficacy 136, 139, BBB penetration 47-50, 76, TME remodeling 47, 49, 55, 57, 60, overcoming heterogeneity 46, 115, and controlled release with reduced toxicity 144. However, several critical research gaps remain to be addressed. 1) Target selection. Many targets, such as EGFRvIII, exhibit unstable expression and are prone to loss following treatment, leading to resistance 31. An ideal target should be closely associated with tumor cell growth, proliferation, and invasion, while maintaining stable expression that is less likely to be lost 192. Therefore, the identification and screening of optimal targets constitute a critical priority, as this will help mitigate off-target effects and reduce the incidence of therapeutic resistance. 2) Biomarker identification and patient stratification. The identification of reliable biomarkers in glioma faces unique challenges compared with many other solid tumors. (a) The presence of the blood-brain barrier (BBB) severely restricts the release of tumor-derived biomolecules into the peripheral circulation, resulting in extremely low abundance of circulating biomarkers, including circulating tumor DNA (ctDNA) and circulating tumor cells (CTCs) 193, 194. Studies have shown that the detection rate of plasma ctDNA in patients with glioma is currently below 20%, which is considerably lower than that reported for other malignancies 194. (b) Glioma exhibits high tumor heterogeneity, making it difficult for a single biopsy or a single biomarker to fully capture the overall tumor status 195. This characteristic often manifests in clinical trials as a lack of statistically significant overall outcomes or statistically significant efficacy only in specific subgroups, suggesting that current patient stratification strategies remain insufficiently refined 49. (c) The TME of glioma is highly immunosuppressive, characterized by a low tumor mutational burden (TMB) and a paucity of high-quality neoantigens, which further limits the identification and application of immune-related biomarkers 196. (d) The expression of certain biomarkers in glioma is dynamic, whereas clinical detection still relies primarily on pathological biopsy, making it difficult to capture real-time changes during treatment 197. (e) Owing to the infiltrative growth pattern of glioma, obtaining tissue samples carries significant risks; although liquid biopsy holds promise, standardized methods for its application have not yet been established 197. 3) Preclinical model selection. The representativeness of certain models is limited. In immunotherapy studies in particular, a fully competent immune system is essential, yet the establishment of humanized models often relies on immunodeficient mice, which compromises the representativeness of efficacy assessments 48. Selecting models that are reproducible, amenable to visualization, biomimetic, and easy to establish is crucial for facilitating clinical translation 198. 4) Drug design. Drug design should take into account patient compliance, prioritizing convenient routes of administration. From the early stages of design, the involvement of pharmaceutical analysis experts is essential to develop analytical protocols that support preparation, purification, and scalable manufacturing. Additionally, aligning drug development with real-world manufacturing processes and patient needs is critical. The establishment of versatile platforms, such as the mSA2 system, can help shorten the research and development cycle and reduce costs 115. 5) Drug development. The safety of CAR-T therapy requires further validation 106-109. Enhancing the persistence of CAR-T cells *in vivo* 112, 114 and preventing the rapid clearance of oncolytic viruses by the host 145 remain key challenges. Currently, glioma immunotherapy continues to be constrained by the BBB, high tumor heterogeneity, the immunosuppressive microenvironment, and adaptive resistance accompanied by tumor evolution 13, 152-157. Future progress is expected through improved delivery routes, combination strategies, the identification of optimal targets, and remodeling of the immune microenvironment. 6) Clinical trial considerations. (a) Patient selection bias. Enrolling patients without disease progression following standard therapy as trial subjects, compared with general patient populations, may lead to an overestimation of treatment efficacy 32. (b) Control arm selection. With the advancement of standard-of-care treatments, OS in historical control groups has improved, making it difficult to reproduce promising results from early-phase trials in subsequent randomized studies 31. (c) Control arm design. Immunotherapy trials should use standard therapy—at minimum TMZ—as the comparator to determine whether the investigational treatment offers improvements over existing therapies 66. (d) Insufficient sample size. The low incidence and high mortality rate of gliomas make patient recruitment challenging, limiting the feasibility of many trials and resulting in underpowered analyses 26, 46, 114, 132. Multicenter international collaboration is urgently needed, along with efforts to standardize treatment protocols. (e) Contradictory findings. Some studies suggest that immunotherapy requires minimal residual disease 46, whereas others have observed long-term survival benefits in patients without complete resection 31. The rindopepimut trial found no correlation between antibody titers and survival benefit, suggesting that T-cell responses may serve as a more relevant selection criterion 31; conversely, most trials, including AFTV, have reported that higher antibody levels correlate with better outcomes 46. 7) Efficacy evaluation. Current imaging modalities are insufficient for assessing treatment response, highlighting the need for novel techniques 139. Certain therapies, such as AFTV, contain theoretically all tumor antigens, making it impossible to evaluate efficacy based on specific antibody titers, necessitating the establishment of new evaluation criteria 46. 8) Clinical translation. The production, quality control, and management of novel biological products require standardization 52. Furthermore, biomarker-guided stratification or individualized treatment approaches are costly and require policy support 7. Although each of the four major immunotherapy strategies faces distinct translational challenges 51, 52, 86, 125, 151, oncolytic virus therapy currently demonstrates the most promising translational potential. This approach has garnered significant attention due to its unique dual mechanism of "oncolysis plus immune activation" 199, 200. G47Δ has been approved in Japan for the treatment of malignant glioma, with a Phase II trial reporting a one-year survival rate of 84.2% and a median OS of 20.2 months, marking a milestone in the field 139. Furthermore, the combination of oncolytic viruses with ICIs has shown synergistic effects in preclinical and early-phase clinical studies, with the potential to convert "cold" tumors into "hot" tumors 134. In contrast, ICIs are unlikely to confer additional survival benefits in the absence of molecular screening or specific biomarker guidance 43, 74, 75. Tumor vaccines are limited in monotherapy due to tumor heterogeneity and antigen loss; however, their value as components of combination regimens should not be overlooked 31. Adoptive cell therapies similarly face challenges such as antigen loss and limited *in vivo* persistence, resulting in modest efficacy as monotherapies 13, 112, 114. Nonetheless, the recently developed mSA2 universal platform offers a promising strategy to address antigen loss by enabling simple exchange of biotinylated antibodies, providing new opportunities for this therapeutic modality 115.

## Figures and Tables

**Table 1:** Summary of key clinical trials of immunotherapy for glioma

Representative Agent/Strategy	Trial Phase	Enrollment (n)	Patient Population	Median OS (months)	Median PFS (months)	Key Efficacy/Survival Endpoint	Key Toxicities/Safety Profile	Reference
Cancer Vaccines								
WT1-mRNA/DC	Phase I/II	40 (13 GBM)	Advanced solid tumors (incl. GBM)	GBM: 43.7	Not reported	• DCR: 69.2% (T1/T2)• ORR: 23.1% (T2)• Type 1 T-cell responses linked to improved OS	• Well tolerated• Local injection site reactions only• No systemic toxicity	21
AV-GBM-1	Phase II	60 (57 treated)	nGBM (pre-RT/TMZ)	16.0	10.7	• PFS 57% longer vs historical controls• 3-yr PFS: 12.3%• 8 doses correlated with OS (HR 0.09, p <0.0001)	• Well tolerated• Local injection site reactions only• No treatment discontinuations	23
ICT-107	Phase II (RCT, DB)	124 (81 vaccine, 43 control)	nGBM (HLA-A1⁺/A2⁺, post-RT/TMZ)	17.0 (vaccine) vs 15.0 (control); p=0.58 (NS)	11.2 (vaccine) vs 9.0 (control); HR 0.57, p=0.011	• PFS significantly improved• HLA-A2 subgroup: greater immune benefit• Methylated HLA-A1: OS 47.6 vs 25.8 mo (p = 0.049)	• Well tolerated; no difference in AEs vs control• Most common: fatigue, convulsions, nausea	25
GSC-DCV	Phase II (RCT, DB)	43 (22 vaccine, 21 placebo)	nGBM/rGBM, post-resection (≥95%)	13.7 (DCV) vs 10.7 (placebo); p=0.05Multivariate HR 2.5, p=0.02	7.7 (DCV) vs 6.9 (placebo); p=0.75	• IDH1ʷᵗTERTᴹᵀ subgroup: OS (p<0.01), PFS (p=0.03)• Low B7-H4 subgroup: OS (p=0.02)	• Well tolerated• Mild fever (1 pt), injection site erythema (1 pt)	26
pp65-DC + DI-TMZ + GM-CSF	Phase I	11	nGBM (post-resection >90%, post-RT/TMZ)	41.1 (vs 19.2 historical, p=0.0001)	25.3 (vs 8.0 historical, p=0.0001)	• 4/11 pts PFS at 59-64 mo• 100% exceeded RPA predicted survival (median gain 30 mo)• pp65 immune response correlated with OS>40 mo (p=0.031)	• Well tolerated• 1 Grade 3 vaccine-related immunologic reaction (GM-CSF sensitization)	29
CMV-ATCT-DC	Phase I/II (RCT, single-blind)	22 enrolled, 15 completed (8 DC, 7 saline)	nGBM (CMV-seropositive, KPS ≥80, post-RT/TMZ)	Not reported (underpowered)	Not reported (underpowered)	• Primary safety endpoint met• Polyfunctional T cells: significant increase in CMV-ATCT-DC vs saline (p=0.0078)• Polyfunctional T cells correlated with OS (R=0.74, p=0.037)	• Well tolerated• No SAEs related to treatment• AEs consistent with standard of care	27
Td toxoid + CMV pp65-DC	Phase I/II (RCT, blinded)	12 (6 Td, 6 DC)	nGBM (CMV-seropositive, KPS ≥80, post-RT/TMZ)	Td: not reached (>36.6)DC: 18.5	Td: not reached (>36.6)DC: 10.8	• DC migration: significant increase in Td vs DC (p=0.049)• Td significantly improved PFS (p=0.013) and OS (p=0.013)• DC migration correlated with survival	• Well tolerated• No vaccine- or Td-related AEs	28
Rindopepimut (ACT III)	Phase II	65	nGBM (EGFRvIII^+^, post-GTR, post-CRT)	21.8 (from study entry)	9.2 (from study entry)	• PFS at 5.5 mo: 66% (primary endpoint met, p=0.017)• 36-mo OS: 26%• 85% ≥4x Ab titer• 67% target loss in evaluable samples	• Grades 1-2 injection site reactions (nearly all)• Grade 3/4 events rare; no fatal AEs	32
Rindopepimut (ACT IV)	Phase III	745 (405 MRD)	nGBM (EGFRvIII^+^, MRD <2 cm² post-CRT)	20.1 (vaccine) vs 20.0 (control); HR 1.01, p=0.93	Not reported	• Primary endpoint not met (futility stopped)• 2-yr OS (SRD): 30% vs 19% (exploratory, p=0.029)	• Grade 3-4: thrombocytopenia (9% vs 6%), fatigue (2% vs 5%), brain edema (2% vs 3%)• Injection site reactions (80% vs 41%, mostly grade 1-2)	31
IDH1-vac	Phase I	33 enrolled, 32 vaccinated	nGBM WHO grade 3/4 IDH1(R132H)^+^, post-RT/TMZ	3-yr OS rate: 0.84	3-yr PFS rate: 0.63	• Immune response rate: 93.3% (across multiple HLA alleles)• PsPD rate: 37.5% (linked to immune response)• MSS correlated with antigen presentation	• No RLT/DLT; AEs grade 1 only• Most common: injection site induration (65.6%), erythema (46.9%)	33
SurVaxM (Phase I)	Phase I	9 enrolled, 8 evaluable	rGBM/AA (survivin⁺, HLA-A*02/03/24)	86.6 weeks (~21.7) (from study entry)	17.6 weeks (~4.4) (from study entry)	• Immune response: 75% (cellular & humoral)• 3 pts PR/SD >6 mo• 7/9 pts survived >12 mo• 1 pt CR, alive NED at 174 weeks	• No SAEs related to vaccine• Mostly grade 1: injection site reactions, fatigue, myalgia• Grade 3 seizure (unrelated)	41
SurVaxM + adj TMZ (Phase IIa)	Phase IIa	64 enrolled, 63 evaluable	nGBM (survivin⁺, HLA-A*02/03/11/24, post-RT/TMZ)	25.9 (from first vaccine)28.4 (from diagnosis)	11.4 (from first vaccine)14.4 (from diagnosis)	• PFS-6 (from diagnosis): 95.2% (primary endpoint met, p<0.0001 vs 54% historical)• 36-mo OS: 41.4%• Ab titer >30,000 correlated with OS (HR 0.41, p = 0.015)• Methylated MGMT: mOS 41.4 mo; Unmethylated: 16.5 mo	• No SAEs related to SurVaxM• Most common: grade 1 injection site reactions (37.5%)• AEs attributable to TMZ (leukopenia, etc.)	34
WT2725	Phase I	62 (20 GBM)	Advanced WT1^+^ malignancies (HLA-A*0201^+^/0206^+^)	Solid tumors: 13.0GBM: 10.2	Solid tumors: ~2GBM: ~2	• irCR (GBM): 13.3% (2/15); irPR: 6.7%• CTL response: dose-dependent (0 mg → 27 mg: 0% → 88.9%)• 2 GBM pts irCR, PFS ≥30.6 and ≥37.0 mo	• No DLT; MTD not reached• Most common: grade 1-2 injection site reactions• No discontinuations	36
IMA950	Phase I	45	nGBM (HLA-A*02^+^, post-resection, receiving CRT+adj TMZ)	15.3 (overall)	Not reported (PFS-6: 74%, PFS-9: 31%)	• Immune response: 90% TUMAP responders• ISR correlated with OS (ISR^+^ mOS 26.7 mo vs ISR^-^ 13.2 mo; HR 0.33, p=0.0001)• MGMT methylated: mOS 28.3 mo vs unmethylated 14.8 mo (p=0.025)	• Well tolerated• Most common: grade 1-2 injection site reactions (26/45)• 2 DLTs (grade 3 fatigue, anaphylaxis)	19
IMA950 + poly-ICLC	Phase I/II	19 (16 GBM, 3 AA)	nGBM/AA (HLA-A2^+^, post-RT/TMZ)	GBM: 19 (from surgery)Whole cohort: 21	GBM: 9.5 (from surgery)PFS-6/9 (GBM): 81%/63%	• Primary immunogenicity endpoint met• CD8 multi-peptide responses: 46.2%• CD4 responses: 84.6%	• Well tolerated• Most common: injection site reactions, fatigue, headache• 1 Grade 4 interstitial pneumonia (unrelated)	37
WT1 cocktail vaccine	Phase I	14 (12 GBM, 2 AA)	rGBM/AA (HLA-A*24:02^+^, WT1^+^, refractory to TMZ)	24.7 weeks (~5.7)	Not reported	• SD at 6 weeks: 43%• 1-year OS rate: 36%• Immune responses: WT1-specific CD8^+^ (64%), CD4^+^ (91%)	• No DLT; No grade 3/4 AEs• Grade 1 injection site reactions (all patients)	38
VEGFRs peptide vaccine + TMZ	Phase I/II	4	nGBM (HLA-A*24:02^+^, post-surgery, receiving RT/TMZ)	Individual: 31.6, 41.8, >46.8, >31.6	Individual: 18.2, 41.8, >46.8, >31.6	• 2/4 patients achieved CR (incl. 1 MGMT unmethylated)• Both CR patients alive at analysis• Histopathological evidence of CTL-mediated killing of tumor vessels, cells, and Tregs	• No grade 3/4 AEs• Grade 1 local skin reaction in 1 patient	39
GAA peptide vaccine + poly-ICLC	Phase I	23	LGG (high-risk), HLA-A2⁺	Not reported	Cohort 1 (no prior RT): 17Cohort 3 (recurrent): 12	• Cohort 1: 91% responded to ≥1 GAA, 82% to ≥3 GAAs• Cohort 3: 56% responded to ≥1 GAA, 33% to ≥3 GAAs• 3 pts in Cohort 1 remain PFS (37, 42, 47+ mo)	• Grade 1-2 injection site reactions (100%)• One case of Grade 3 fever/fatigue (RLT)	40
Personalized mutIDH1 multi-peptide vaccine	Phase I/II	52	IDH1-mutant glioma (grade 2-4), newly diagnosed or recurrent	Not reached (median f/u 36 mo)	Not reported	• mutIDH1 immune response: 89%• Immunological responders had significantly longer OS (HR 0.09; p=0.0057)• mutIDH1-specific CD4^+^ T cells were polyfunctional and polyclonal• Identical TCRβ sequences found in patients with shared HLA-DQ alleles	• Well tolerated• Grade 1 injection site reactions only• No grade ≥3 AEs	20
Personalized peptide vaccination (PPV) based on pre-existing IgG levels	Phase III (RCT, DB)	88 (58 PPV, 30 placebo)	HLA-A24^+^ recurrent GBM, refractory to TMZ/RT	PPV: 8.4Placebo: 8.0 (p=0.62)	Not shown (no significant difference)	• Primary endpoint not met• PPV detrimental with SART2-93 peptide (HR 15.9), age ≥70 yr (HR 7.9), weight >70 kg (HR 4.1), PS3 (HR 2.8)• PPV beneficial without SART2-93 + age <70 yr (HR 0.49, p=0.03)• Lower baseline CD11b^+^CD14^+^HLA-DRlow monocytes and higher CD3^+^CD4^+^CD45RA^-^ T cells correlated with improved OS	• Well tolerated• Most common: injection site reactions (PPV 71%, placebo 53%)• Grade ≥3 AEs: PPV 40%, placebo 37%• One grade 3 pulmonary embolism (PPV-related)	44
Autologous formalin-fixed tumor vaccine (AFTV)	Phase IIb (RCT, DB, placebo-controlled)	57 (30 AFTV, 27 placebo)	Newly diagnosed supratentorial GBM, age 16-75 yr, KPS ≥60%	AFTV: 25.6Placebo: 31.5 (p=0.64)	AFTV: 13.3Placebo: 13.3 (p=0.98)	• Primary endpoint not met• Total resection subgroup: 3-yr OS 80% vs 54% (p = 0.16); 3-yr PFS 81% vs 46% (p = 0.067)• p53-negative subgroup: 3-yr OS 79% vs 43% (p = 0.072)• DTH-2 response not associated with survival• Met predefined criteria for Phase III trial	• Well tolerated; no significant difference in AEs• Grade ≥3 AEs: AFTV 23%, placebo 30%• No serious AEs attributed to AFTV	46
APVAC1 + APVAC2 + poly-ICLC + GM-CSF	Phase I	16 enrolled, 15 vaccinated	nGBM (HLA-A*02:01⁺/24:02⁺, post-GTR, receiving CRT)	29.0 (n=15 evaluable)	14.2	• APVAC1 immune response: 92.3%• APVAC2 immune response: 80% (Th1-type CD4^⁺^)• Low baseline Tregs correlated with higher memory cell induction	• Well tolerated• Most common: grade 1-2 injection site reactions• Two cases of anaphylaxis (potentially GM-CSF-related)• One grade 3 cerebral edema	45
Immune Checkpoint Inhibitors								
Ipilimumab + Nivolumab + adj TMZ	Phase I	32 enrolled, 31 evaluable	nGBM (unifocal, supratentorial, post-GTR/NTR, post-CRT)	Combination cohort (n=19): 20.7 (from treatment start)	Combination cohort (n=19): 16.1	• Primary safety endpoint met: 1 DLT in Cohort 1, 1 DLT in Cohort 2, 0 DLTs in Cohort 3• 1-year OS rate (combination): 68.4%• 2-year OS rate (combination): 31.6%	• Well tolerated; no Grade 5 AEs• No unexpected toxicity• Most common Grade 3-4: skin disorders (38.5%), lab abnormalities (23.1%)	42
Ipilimumab + Nivolumab + RT (no TMZ)	Phase II/III (RCT)	159 (79 immunotherapy, 80 TMZ)	nGBM (MGMT-unmethylated, KPS ≥70, post-resection)	Immature (~13 mo each)HR 0.95, p=0.36	7.7 (immunotherapy) vs 8.5 (TMZ)HR 1.47, p=0.96	• Primary endpoint not met (PFS not improved)• Trial terminated• 2 treatment-related deaths on immunotherapy arm (autoimmune disorder, colitis)	• No significant difference in AEs between arms (p = 0.69)• Grade ≥3 AEs: 37.8% (TMZ) vs 41.0% (immunotherapy)• Grade 5 events: 0 vs 2	43
Atezolizumab + RT/TMZ	Phase I/II	60	nGBM (WHO 2016 criteria, KPS ≥60)	All pts: 18.0IDH-wt: 16.1MGMT methylated: 25.4MGMT unmethylated: 14.6	Not reported	• ORR: 23.3% (CR 10% + PR 13.3%)• DoR: 13.91 mo• High ESTIMATE Immune Score tumors: mOS 24.8 vs 14.5 mo (HR 0.45, p = 0.02)• Mesenchymal subtype: mOS 26.5 mo	• Well tolerated; no new safety signals• Grade ≥3 treatment-related AEs: 56.7% (most common: lymphopenia)• 3 patients removed due to hepatitis/pneumonitis (DLTs)	70
Nivolumab + Ipilimumab (pre-radiation)	Phase I	15	nGBM (Grade 3/4, post-surgery, pre-RT)	19.3 (95% CI, 12.9-NA)	1.3 (95% CI, 0.92-2.99)	• Feasibility demonstrated (treatment started median 38 days post-op)• MGMT methylated: mOS 35.7 mo vs unmethylated: 12.6 mo (p = 0.004)• Paired tumor analysis (n = 5): post-progression tumors showed ↑ TGF-β/ERBB, ↓ PPAR signaling, ↑ monocytes, ↓ B cells.• Growing tumors trended toward higher baseline LAG-3.	• Well tolerated; no DLTs• Most common: rash, pruritus, fatigue, nausea, anorexia• Grade 3: lipase ↑ (2), anorexia (1), pruritus (1), rash (3)• One Grade 4 cerebral edema• No Grade 5 events	68
Neoadjuvant + Adjuvant Pembrolizumab	Phase II (single-arm expansion)	25 (expansion) + 32 (initial) = 57 pooled	rGBM (surgically accessible, 1st/2nd relapse, KPS ≥70)	Expansion: 6.8Initial neoadjuvant: 13.7Initial adjuvant: 7.5	Expansion: 2.5Initial neoadjuvant: 3.3Initial adjuvant: 2.5	• Primary pharmacodynamic endpoint met: Cell cycle gene signature decreased in neoadjuvant vs adjuvant tumors (p=0.009)• IFN-high/CC-low subgroup (n=13): mOS 13.8 mo• No confirmed OS benefit for neoadjuvant regimen	• Well tolerated; no new safety signals• Grade ≥3 AEs in 6 pts: cerebral edema (2), headache, fatigue, adrenal insufficiency, hyperthyroidism	69
Tislelizumab + Low-Dose Bevacizumab	Phase II	32	rGBM	14.3	8.2	• ORR: 56.3%• 12-month OS: 43.8%• >20% reduction in MAF/TMB post-treatment correlated with improved PFS and OS (p = 0.0005-0.008)• Baseline MUC16 mut, H3F3B amp, SRSF2 amp associated with worse OS	• Any grade AEs: 90.6% (most common: anemia, fatigue, increased ALT)• Grade ≥3: 6.2% (incl. one grade 4 acute pancreatitis)• No grade 5 events	73
Adoptive Immune Cell Therapy								
CART-EGFRvIII cells + Pembrolizumab	Phase I	7	nGBM (EGFRvIII^+^, MGMT-unmethylated)	11.8 (90% CI, 9.2-14.2)	5.2 (90% CI, 2.9-6.0)	• Primary safety endpoint met: no DLTs• Antigen reduction: EGFRvIII decreased post-treatment in 6/7 pts• ↑ IFN-stimulated T cells in post-treatment tumors correlated with time from relapse to death (Spearman p=0.03)	• Safe and well-tolerated• No CRS, no ICANS• One patient experienced severe (grade 3-4) immune-related AE (liver/kidney injury) likely related to Pembrolizumab	13
CART-EGFRvIII cells (single infusion)	Phase I	10	rGBM (EGFRvIII^+^, MGMT-unmethylated)	251 days (~8)	Not evaluable	• Primary safety & feasibility endpoint met• On-target activity: EGFRvIII expression decreased in 5/7 assessed pts (p = 0.03)• Trafficking: Higher CAR T concentration in brain vs blood in 2 pts• TME changes: ↑ immunosuppressive molecules (IDO1, PD-L1, FoxP^3^, IL-10)	• No EGFR-directed toxicity, no systemic CRS, no neurotoxicity typical of CD19-CARs• 3 subjects with clinically significant neurologic events (possibly related to disease or local inflammation)• No DLTs	14
CART-EGFR-IL13Rα2 (bivalent, ICV)	Phase I	18	rGBM (EGFR-amplified, IDH-wt)	Not reached (f/u 8.1 mo)	1.9 (90% CI, 1.1-3.4)	• ORR: 8% (1/13)• Tumor regression: 62% (8/13)• Durable disease: 2 pts PFS >6 mo (7.7, 16.6)• MTD/RDE: 2.5 × 10⁷ cells	• CRS: 100% (all grades 1-2)• Grade 3 neurotoxicity: 56% (no grade 4-5)• One DLT (grade 3 anorexia, weakness, fatigue) at MTD	110
IL-13(E13Y)-zetakine CD8+ CTLs	Phase I	3	rGBM	Not reported (mean ~11)	Not reported	• Feasibility demonstrated• Antigen loss: In one patient, IL13Rα2 expression decreased post-treatment• Radiographic response: In one patient, ↑ necrotic volume on MRI post-retreatment	• Well tolerated at 10⁷ and 5×10⁷ cell doses• At 10⁸ cell dose: Grade 3 headache (1 pt), Grade 3 neurological event (shuffling gait, tongue deviation; 1 pt); both transient and manageable	111
GRm13Z40-2 cells (allogeneic, GR-knockout)	Phase I	6	rGBM (unresectable, IL13Rα2^+^, on dexamethasone)	2.9 (post-treatment)	Not reported	• Proof-of-Concept: First-in-human allogeneic, ZFN-modified, steroid-resistant CAR T product• Anti-tumor activity: Transient tumor reduction/necrosis at infusion site in 4/6 pts	• Well tolerated• All treatment-related AEs ≤ grade 3, transient, manageable• No DLTs, GVHD, or severe treatment-related neuroinflammation	112
GD2-specific 4SCAR-T (IV ± intracavitary)	Phase I	8	rGBM (GD2^+^, IDH-wt; 4 adults, 4 children)	10 (range, 3-24) from infusion	Not reported	• ORR: 50% (4/8 PR, 3-24 mo duration)• Disease control (PR+SD): 62.5%• Antigen loss & TME modulation: ↓ GD2, ↑ CD8^+^, ↓ CD163^+^ M2 macrophages (1 pt)	• Safe and well-tolerated• No severe AEs (grade ≥4) or CAR-T-related neurotoxicity• One patient: grade 2 seizures and grade 3 headache probably related to infusion	113
CLTX-CAR T cells (intracavitary/intratumoral)	Phase I (Interim)	4	rGBM (MMP-2^+^, heavily pretreated)	<6 (3 pts)1 pt >20	Not reported	• Disease Control Rate: 75% (3/4 SD)• Regional control: No recurrence at infusion site for >9 weeks (1 pt)• Proof-of-Concept: First-in-human toxin-based CAR platform	• Well tolerated; no DLTs or CRS• No grade ≥3 CNS events attributed as probable/definite• Grade 3 cerebral edema (possibly related) in 2 pts• No humoral immunogenicity detected	114
Oncolytic Virotherapy								
MV-CEA	Phase I	22	rGBM (anti-MV immune at baseline)	11.6	3.4	• 12-month OS: 45.5% (favorable vs historical controls)• Disease control rate (SD): 88.9% (Group A), 92.3% (Group B)• Biomarker (ISG signature): DLDA score inversely correlated with viral replication (R=-0.6, p=0.04)• TME remodeling: ↑ CD8^+^ and CD68^+^ infiltration (day 5)	• Well tolerated; no DLTs up to max dose (2×10⁷ TCID50)• Only grade 1-2 AEs• No viremia, viral shedding, or treatment-induced immunosuppression	136
DNX-2401 + Pembrolizumab	Phase I/II	49	rGBM/rGS (1st/2nd recurrence, unresected)	12.5	3.4	• Primary ORR endpoint (10.4%) not met (p=NS vs 5%)• Secondary 12-mo OS endpoint (52.7%) met (vs 20% control)• Clinical benefit rate (SD or better): 56.2%• 3 patients alive at 45, 48, and 60 mo• Biomarker: Responses only in the "TMEmedium" subtype (exploratory)	• Well tolerated; no DLTs• Most common: brain edema (37%), headache (31%), fatigue (29%)• Grade 3 AEs: 22%; one grade 4 event• No treatment-related deaths	132
Ad-TD-nsIL12	Phase I	8	rHGG (with ventricular system connection)	5.1 (range, 3.1-21.2) from first injection	Not reported	• Primary MTD endpoint: 1×10¹⁰ vp (grade 3 seizures as DLT at 5×10¹⁰ vp)• Antitumor activity: 1 CR, 1 PR (both in Cohort 2)• TME remodeling: ↑ CD4^+^/CD8^+^ T cell infiltration, confirmed viral replication	• Well tolerated at ≤1×10¹⁰ vp• Most common: fever, cognitive disturbance, nausea/vomiting• Grade 3 seizures (DLTs) at highest dose in 2/2 pts• No viral DNA in blood/CSF	137
NSC-CRAd-S-pk7 (neural stem cell-loaded)	Phase I	12	nGBM (WHO grade III/IV)	18.4	9.05	• Primary safety endpoint met: No DLT up to highest dose• MGMT unmethylated subgroup (exploratory): mOS 18.0 mo, mPFS 8.8 mo (favorable vs historical)• TME remodeling: ↑ CD8^+^ T cell infiltration, ↑ PD-1 expression, ↓ survivin/syndecan-1	• Safe and well-tolerated; no DLT• One patient: grade 3 viral meningitis (probably related, due to inadvertent intraventricular injection)• No treatment-related deaths• RP2D: 1.50×10⁸ NSCs / 1.875×10¹¹ vp	138
G47Δ (triple-mutated HSV-1)	Phase II	19	Residual/rGBM (post-RT/TMZ)	20.2 (from 1st injection)28.8 (from initial surgery)	4.7 (from 1st injection)	• Primary endpoint (1-yr OS): 84.2% (met, trial terminated early)• TME remodeling: ↑ CD4^+^/CD8^+^ TILs with repeated dosing, low Foxp3^+^ Tregs• Regulatory outcome: First approved oncolytic virus for malignant glioma (Japan, 2021)	• Safe and well-tolerated; no DLT• Most common: fever (89.5%), vomiting (57.9%), nausea (52.6%)• Grade ≥3 lymphocytopenia: 26.3% (resolved without treatment)	139
G47Δ (triple-mutated HSV-1)	Phase I/II	13	rGBM (post-RT/TMZ)	7.3 (from last dose)30.5 (from initial surgery)	8 days (from last dose)	• 1-year OS rate (from last dose): 38.5%• Long-term survival: 3 patients >46 mo• Best overall response (2-yr, exploratory): CR 7.7%, PR 7.7%• TME remodeling: ↑ CD4^+^/CD8^+^ T cell infiltration post-treatment	• Safe and well-tolerated• Most common: fever (61.5%), headache (61.5%), vomiting (46.2%)• Grade 3 events: leukopenia, headache, vomiting, neurological disorder (few pts)• No DLT	140

Abbreviations: AA, anaplastic astrocytoma; Ab, antibody; adj, adjuvant; AE, adverse event; AFTV, autologous formalin-fixed tumor vaccine; ALT, alanine aminotransferase; amp, amplification; APVAC, actively personalized vaccination; CAR, chimeric antigen receptor; CBR, clinical benefit rate; CI, confidence interval; CMV, cytomegalovirus; CNS, central nervous system; CR, complete response; CRT, chemoradiotherapy; CRS, cytokine release syndrome; CSF, cerebrospinal fluid; CTL, cytotoxic T lymphocyte; DB, double-blind; DC, dendritic cell; DCR, disease control rate; DI-TMZ, dose-intensified temozolomide; DLT, dose-limiting toxicity; DLDA, diagonal linear discriminant analysis; DoR, duration of response; DTH, delayed-type hypersensitivity; dx, diagnosis; ECOG, Eastern Cooperative Oncology Group; EGFR, epidermal growth factor receptor; EGFRvIII, epidermal growth factor receptor variant III; ESTIMATE, Estimation of Stromal and Immune cells in Malignant Tumors using Expression data; f/u, follow-up; FACS, fluorescence-activated cell sorting; FoxP3, forkhead box P3; GAA, glioma-associated antigen; GBM, glioblastoma; GM-CSF, granulocyte-macrophage colony-stimulating factor; GR, glucocorticoid receptor; GSC-DCV, glioma stem cell-derived dendritic cell vaccine; GTR, gross total resection; GVHD, graft-versus-host disease; H3F3B, H3.3 histone B; HLA, human leukocyte antigen; HR, hazard ratio; HSV, herpes simplex virus; ICANS, immune effector cell-associated neurotoxicity syndrome; ICB, immune checkpoint blockade; ICI, immune checkpoint inhibitor; ICV, intracerebroventricular; IDH, isocitrate dehydrogenase; IDO1, indoleamine 2,3-dioxygenase 1; IFN, interferon; Ig, immunoglobulin; IHC, immunohistochemistry; IL, interleukin; incl., including; IQR, interquartile range; irCR, immune-related complete response; irPR, immune-related partial response; iRANO, immunotherapy Response Assessment in Neuro-Oncology; ISG, interferon-stimulated gene; ISR, IMA950-specific response; ITT, intent-to-treat; IV, intravenous; KPS, Karnofsky Performance Status; LAG-3, lymphocyte activation gene 3; LGG, low-grade glioma; MAF, mutant allele fraction; MGMT, O⁶-methylguanine-DNA methyltransferase; mo, months; MRS, magnetic resonance spectroscopy; MRD, measurable residual disease; MSS, mutation-specificity score; MTD, maximum tolerated dose; MUC16, mucin 16; mut, mutation; MV, measles virus; MVAF, maximal somatic variant allelic frequency; NA, not available; NED, no evidence of disease; nGBM, newly diagnosed glioblastoma; NR, not reached; NS, not significant; NTR, near total resection; ORR, objective response rate; OS, overall survival; PD, progressive disease; PD-1, programmed cell death protein 1; PD-L1, programmed death-ligand 1; PFS, progression-free survival; pfu, plaque-forming units; PLR, platelet-to-lymphocyte ratio; poly-ICLC, polyinosinic-polycytidylic acid stabilized with poly-L-lysine and carboxymethylcellulose; post-, post-treatment; PPV, personalized peptide vaccination; pre-, pre-treatment; PR, partial response; PsPD, pseudoprogression; PS, performance status; pt, patient; RANO, Response Assessment in Neuro-Oncology; RCT, randomized controlled trial; RDE, recommended dose for expansion; rGBM, recurrent glioblastoma; rGS, recurrent gliosarcoma; rHGG, recurrent high-grade glioma; RLT, restricted limiting toxicity; RPA, recursive partitioning analysis; RP2D, recommended Phase 2 dose; RT, radiotherapy; SAE, serious adverse event; SD, stable disease; SRD, significant residual disease; SRSF2, serine and arginine rich splicing factor 2; T1, time point 1; T2, time point 2; TCID50, 50% tissue culture infectious dose; Td, tetanus-diphtheria; TERT, telomerase reverse transcriptase; TGF-β, transforming growth factor beta; TIL, tumor-infiltrating lymphocyte; TISF, tumor *in situ* fluid; TMB, tumor mutational burden; TME, tumor microenvironment; TMZ, temozolomide; TNF, tumor necrosis factor; Treg, regulatory T cell; TUMAP, tumor-associated peptide; VEGF, vascular endothelial growth factor; VEGFR, vascular endothelial growth factor receptor; vp, viral particles; vs, versus; wk, week; WT1, Wilms tumor 1; yr, year; ZFN, zinc finger nuclease.

**Table 2:** Ongoing clinical trials of immunotherapy combination strategies in glioma

Combination Strategy	Specific Regimen	Trial Phase	Target Population	Primary Endpoint / Objective	NCT Number
Vaccine + Chemoradiotherapy	Tamavaq™ personalized neoantigen vaccine + Radiotherapy + Temozolomide	Early Phase I	Newly diagnosed glioma	Primary: Safety, efficacy	NCT07077616
Immuno-immuno combination	Autologous TIL therapy + Pembrolizumab (anti-PD-1)	Phase I/II	Advanced brain cancer (gliomas, meningiomas, etc.)	Primary: Efficacy, safety	NCT06640582
Immunotherapy + Chemotherapy	Tarlatamab (DLL3-targeted BiTE) + Metronomic Temozolomide	Phase I/II	Adolescents and adults with recurrent high-grade CNS tumors (including IDH-mutant glioma, ependymoma, medulloblastoma)	Primary: Safety; Secondary: Clinical activity	NCT07243470
Immunotherapy + Anti-angiogenic	CTX-009 (DLL4/VEGF-A bispecific antibody) + CTX-471 (anti-CD137 agonist antibody)	Phase Ib/II	Recurrent glioblastoma (CNS WHO grade 4, IDH-wildtype)	Primary: Safety; Secondary: Efficacy (preliminary)	NCT07392957
Dendritic cell vaccine + Standard of care	DOC1021 (autologous dendritic cell immunotherapy) + Radiotherapy + Temozolomide + Tumor Treating Fields (TTFields)	Phase II	Newly diagnosed glioblastoma (WHO grade 4)	Primary: Safety, efficacy	NCT06805305
Peptide vaccine + Chemotherapy	PEP-CMV vaccine + Adjuvant temozolomide (one cycle)	Phase I	Newly diagnosed MGMT-unmethylated glioblastoma	Primary: Safety, tolerability	NCT06132438
ICI + Anti-angiogenic + Radiotherapy	Retifanlimab (anti-PD-1) + Bevacizumab + Hypofractionated Radiotherapy	Phase II	Recurrent glioblastoma	Primary: Efficacy	NCT06160206
Gene therapy + Immunotherapy + Surgery	TGX-007 (AAV-HSV-tk + IL-12) intratumoral injection + oral valacyclovir + standard of care surgery	Phase I/II	Newly diagnosed or recurrent glioblastoma	Primary: Safety, OBD; Secondary: Efficacy	NCT07346144
CAR-T+Chemotherapy	Autologous GD2-C7R CAR-T cells (GD2 CAR + constitutively active IL-7 receptor) + Cyclophosphamide/Fludarabine lymphodepletion	Phase I	GD2-expressing Brain Tumors (DMG, HGG, DIPG, medulloblastoma)	Primary: MTD; Secondary: CAR-T persistence, anti-tumor activity	NCT04099797
Dual ICI + Surgery	Ipilimumab (anti-CTLA-4) + Nivolumab (anti-PD-1) administered IV (neo-adjuvant) and intracranial (adjuvant via Ommaya reservoir) + maximal safe resection	Phase I	Resectable recurrent glioblastoma	Primary: Safety, feasibility	NCT06097975
Bispecific antibody-armed T cells + Focused ultrasound	EGFR BATs (anti-CD3 × anti-EGFR bispecific antibody-armed autologous T cells) + Low-intensity focused ultrasound (LIFU) with microbubbles for BBB opening	Phase I	Newly diagnosed MGMT unmethylated IDH-wildtype glioblastoma	Primary: Safety, feasibility; Secondary: T cell trafficking	NCT07343986
bispecific antibody-immunotoxin + immunomodulator	D2C7-IT (bispecific antibody) + 2141-V11 (immunomodulator)	Phase I/II	Resected recurrent glioblastoma	Primary: Safety; Secondary: Efficacy	NCT06455605
Radioimmunotherapy + ICI	Actimab-A (anti-CD33 monoclonal antibody conjugated with actinium-225) + Cemiplimab (anti-PD-1)	Phase I	Recurrent glioblastoma	Primary: Safety, tolerability; Secondary: Efficacy	NCT07422363
Allogeneic γδ T cell therapy ± Chemo/Radiotherapy	Allogeneic Vγ9Vδ2 T cells (intraventricular via Ommaya reservoir) ± Temozolomide or radiotherapy	Phase I	WHO grade IV malignant glioma (glioblastoma, DIPG, medulloblastoma, ependymoma)	Primary: Safety, feasibility; Secondary: Efficacy	NCT06396481
Bispecific antibody-immunotoxin+ immunomodulator + Radiotherapy	D2C7-IT (bispecific antibody-immunotoxin) + 2141-V11 (immunomodulator)	Phase I/II	Newly diagnosed MGMT unmethylated glioblastoma	Primary: Safety; Secondary: Efficacy	NCT05734560
CAR-T + Dual ICIs + Neoadjuvant/Adjuvant	IL13Rα2-CAR T cells (intracranial via Rickham catheter) + Nivolumab (anti-PD-1) ± Ipilimumab (anti-CTLA-4)	Phase I	Resectable recurrent glioblastoma	Primary: Safety; Secondary: Efficacy	NCT04003649
IL-7 agonist + PD-1 inhibitor + Surgery	Neoadjuvant Efineptakin Alfa (NT-I7) and Pembrolizumab	Phase II	Recurrent glioblastoma	Primary: Safety; Secondary: Efficacy	NCT05465954
PD-L1 inhibitor + Multi-kinase inhibitor	Atezolizumab (anti-PD-L1) + Cabozantinib (VEGFR/MET/AXL inhibitor)	Phase I/II	Recurrent glioblastoma	Primary: Safety; Secondary: Efficacy	NCT05039281
Dendritic cell vaccine + PD-1 inhibitor	Hybrid dendritic cell vaccine + Pembrolizumab (anti-PD-1)	Phase III	Adult glioblastoma (post standard-of-care surgery, chemotherapy, radiotherapy)	Primary: Efficacy, safety	NCT06749925
Dual ICIs + Chemotherapy + Ultrasound BBB opening	Balstilimab (anti-PD-1) + Botensilimab (anti-CTLA-4) + Liposomal doxorubicin + Soncloud-9 ultrasound device for BBB opening	Phase IIa	Newly diagnosed glioblastoma (post-radiotherapy)	Primary: Safety, feasibility; Secondary: Efficacy	NCT05864534
PD-L1/VEGF bispecific antibody + Chemotherapy	BNT327 (Pumitamig, anti-PD-L1 x VEGF-A bispecific antibody) ± Temozolomide	Phase II	Recurrent glioblastoma (WHO grade IV, post-radiotherapy + Temozolomide)	Primary: Safety; Secondary: Efficacy	NCT07297212
CAR-NK + IL-15 agonist + Anti-VEGF + TTFields	Nogapendekin alfa inbakicept (IL-15 agonist) + PD-L1 t-haNK (CAR-NK cells) + Bevacizumab + Tumor Treating Fields (TTFields)	Phase II/IIB	Recurrent or progressive glioblastoma	Primary: Safety; Secondary: Efficacy	NCT06061809
Allogeneic virus-specific T cells + PD-1 inhibitor	Allogeneic CMV-specific T cells + Pembrolizumab (anti-PD-1)	Phase I/II	Newly diagnosed and recurrent glioblastoma/grade 4 astrocytoma	Primary: MTD, RP2D; Secondary: Anti-tumor activity	NCT06157541

Abbreviations: AAV, adeno-associated virus; anti-CD137, anti-cluster of differentiation 137; anti-CTLA-4, anti-cytotoxic T-lymphocyte-associated protein 4; anti-PD-1, anti-programmed cell death protein 1; anti-PD-L1, anti-programmed death-ligand 1; AXL, AXL receptor tyrosine kinase; BBB, blood-brain barrier; BiTE, bispecific T-cell engager; CAR, chimeric antigen receptor; CAR-NK, chimeric antigen receptor natural killer; CAR-T, chimeric antigen receptor T-cell; CMV, cytomegalovirus; CNS, central nervous system; DIPG, diffuse intrinsic pontine glioma; DLL3, delta-like ligand 3; DLL4, delta-like ligand 4; DMG, diffuse midline glioma; EGFR, epidermal growth factor receptor; GD2, disialoganglioside 2; HGG, high-grade glioma; HSV-tk, herpes simplex virus thymidine kinase; ICI, immune checkpoint inhibitor; IDH, isocitrate dehydrogenase; IL, interleukin; IL13Rα2, interleukin-13 receptor alpha 2; LIFU, low-intensity focused ultrasound; MET, mesenchymal-epithelial transition factor; MGMT, O⁶-methylguanine-DNA methyltransferase; MTD, maximum tolerated dose; OBD, optimal biological dose; PD-1, programmed cell death protein 1; PD-L1, programmed death-ligand 1; RP2D, recommended Phase II dose; TIL, tumor-infiltrating lymphocyte; TTFields, tumor treating fields; VEGF, vascular endothelial growth factor; VEGF-A, vascular endothelial growth factor A; VEGFR, vascular endothelial growth factor receptor.

**Table 3:** Predictive biomarkers for therapeutic response

Therapeutic Strategy	Biomarker	Predictive Role	Corresponding Study
Cancer Vaccine	HLA-A2, HLA-A1	HLA typing for patient selection	ICT-107 25
	IDH1, TERT, B7-H4	IDH1ʷᵗTERTᴹᵀ subgroup showed OS/PFS benefit; low B7-H4 subgroup showed OS benefit	GSC-DCV 26
	MGMT methylation	Methylated patients: mOS 41.4 months vs 16.5 months in unmethylated	SurVaxM + adj TMZ 34
	MGMT methylation	Among ISR⁺ patients, methylated: mOS 28.3 months vs 14.8 months in unmethylated	IMA950 19
	HLA-A*24:02, WT1	Patient selection based on HLA typing and WT1 expression	WT1 cocktail vaccine 38
	HLA-A*24:02	Patient selection based on HLA typing	VEGFRs peptide vaccine + TMZ 39
	IDH1 mutation	Targeting IDH1-mutant glioma patients	Personalized mutIDH1 multi-peptide vaccine 20
Immune Checkpoint Inhibitor	MGMT methylation	Methylated patients: mOS 35.7 months vs 12.6 months in unmethylated	Nivolumab + Ipilimumab (pre-radiation) 68
CAR T-Cell Therapy	EGFRvIII, MGMT unmethylated	Patient selection based on EGFRvIII expression; MGMT unmethylated status for patient selection	CART-EGFRvIII + Pembrolizumab 13; CART-EGFRvIII cells 14
	EGFR amplification, IDH wild-type	Patient selection based on EGFR amplification and IDH wild-type status	CART-EGFR-IL13Rα2 110
	IL13Rα2	Patient selection based on IL13Rα2 expression	GRm13Z40-2 cells 112
	GD2, IDH wild-type	Patient selection based on GD2 expression; IDH wild-type	GD2-specific 4SCAR-T 113
	MMP-2	Patient selection based on MMP-2 expression	CLTX-CAR T cells 114
Oncolytic Virotherapy	ISG signature	ISG signature score inversely correlated with viral replication, predicting treatment response	MV-CEA 136
	TME^medium^ subtype	Treatment response observed only in the "TME^medium^" subtype (exploratory)	DNX-2401 + Pembrolizumab 132

Abbreviations: adj TMZ, adjuvant temozolomide; B7-H4, B7 homolog 4; CAR-T, chimeric antigen receptor T-cell; EGFR, epidermal growth factor receptor; EGFRvIII, epidermal growth factor receptor variant III; GD2, disialoganglioside 2; GSC-DCV, glioma stem cell-derived dendritic cell vaccine; HLA, human leukocyte antigen; IDH, isocitrate dehydrogenase; IL13Rα2, interleukin-13 receptor alpha 2; ISG, interferon-stimulated gene; ISR, immune response; MGMT, O⁶-methylguanine-DNA methyltransferase; MMP-2, matrix metalloproteinase-2; mOS, median overall survival; OS, overall survival; PFS, progression-free survival; TERT, telomerase reverse transcriptase; TME, tumor microenvironment; TMZ, temozolomide; VEGFR, vascular endothelial growth factor receptor; WT1, Wilms tumor 1.

**Table 4:** Biomarker guiding combination therapy selection

Therapeutic Strategy	Biomarker	Predictive Role	Corresponding Study
Cancer Vaccine	MGMT methylation	Methylated patients: mOS 41.4 months vs 16.5 months in unmethylated	SurVaxM + adj TMZ 34
	MGMT methylation	Among ISR⁺ patients, methylated: mOS 28.3 months vs 14.8 months in unmethylated	IMA950 19
Immune Checkpoint Inhibitor	MGMT methylation	Methylated patients: mOS 35.7 months vs 12.6 months in unmethylated	Nivolumab + Ipilimumab (pre-radiation) 68
	ESTIMATE Immune Score, molecular subtype	High immune score tumors: mOS 24.8 vs 14.5 months; mesenchymal subtype: mOS 26.5 months	Atezolizumab + RT/TMZ 70
	PLR, MUC16, H3F3B, SRSF2	Low PLR correlated with improved PFS/OS; MUC16 mutation, H3F3B amplification, SRSF2 amplification associated with worse OS	Tislelizumab + Low-Dose Bevacizumab 72, 73
CAR T-Cell Therapy	EGFRvIII, MGMT unmethylated	Patient selection based on EGFRvIII expression; MGMT unmethylated status for patient selection	CART-EGFRvIII + Pembrolizumab 13; CART-EGFRvIII cells 14
	EGFR amplification, IDH wild-type	Patient selection based on EGFR amplification and IDH wild-type status	CART-EGFR-IL13Rα2 110
	IL13Rα2	Patient selection based on IL13Rα2 expression	GRm13Z40-2 cells 112
Oncolytic Virotherapy	ISG signature	ISG signature score inversely correlated with viral replication, predicting treatment response	MV-CEA 136
	TME^medium^ subtype	Treatment response observed only in the "TME^medium^" subtype (exploratory)	DNX-2401 + Pembrolizumab 132

Abbreviations: adj TMZ, adjuvant temozolomide; CAR-T, chimeric antigen receptor T-cell; EGFR, epidermal growth factor receptor; EGFRvIII, epidermal growth factor receptor variant III; ESTIMATE, Estimation of Stromal and Immune cells in Malignant Tumors using Expression data; H3F3B, H3.3 histone B; IDH, isocitrate dehydrogenase; IL13Rα2, interleukin-13 receptor alpha 2; ISG, interferon-stimulated gene; ISR, immune response; MGMT, O⁶-methylguanine-DNA methyltransferase; MMP-2, matrix metalloproteinase-2; mOS, median overall survival; MUC16, mucin 16; OS, overall survival; PFS, progression-free survival; PLR, platelet-to-lymphocyte ratio; RT, radiotherapy; SRSF2, serine and arginine rich splicing factor 2; TME, tumor microenvironment; TMZ, temozolomide.

## Abbreviations

AEs: Adverse Events
AFTV: Autologous Formalin-fixed Tumor Vaccine
ARMed CAR-T: Checkpoint Antibody Receptor-modified Chimeric Antigen Receptor T-cell
ARPC1B: Actin Related Protein 2/3 Complex Subunit 1B
ATA: Autologous Tumor Antigen
BBB: Blood-Brain Barrier
BRD9: Bromodomain-containing Protein 9
CAR-T: Chimeric Antigen Receptor T-cell
CFZ: Clofazimine
CNS: Central Nervous System
DAMPs: Damage-associated molecular patterns
DDR: DNA Damage Response
DFMO: Difluoromethylornithine
DCs: Dendritic Cells
dsRNA: Double-stranded RNA
EGFRvIII: Epidermal Growth Factor Receptor Variant III
EV: Extracellular Vesicles
GAA: Glioma-associated Antigen
GAPVAC: Glioma Active Personalized Vaccine Consortium
GARP: Glycoprotein A Repetitions Predominant
GBM: Glioblastoma
GBO: Glioblastoma Organoid
GSC: Glioma stem cell
HCMV: Human Cytomegalovirus
HLA-A2: Human Leukocyte Antigen A2
HSV-1: Herpes Simplex Virus Type 1
IBDV: Infectious Bursal Disease Virus
ICB: Immune Checkpoint Blockade
ICD: Immunogenic Cell Death
ICIs: Immune Checkpoint Inhibitors
IDH1: Isocitrate Dehydrogenase
IDH1/2: Isocitrate Dehydrogenase 1/2
IDH1-vac: Isocitrate Dehydrogenase 1 Mutation-targeted Vaccine
ISG: Interferon-stimulated Gene
IV: intravenous delivery
JAK: Janus Kinase
KLH: Keyhole Limpet Hemocyanin
LAK: Lymphokine-activated Killer
LNPs: Lipid Nanoparticles
MCP: Multi-cationic Protein
MDSCs: Myeloid-derived Suppressor Cells
METTL14: Methyltransferase-like 14
MGMT: O-6-Methylguanine-DNA Methyltransferase
MHC: Major histocompatibility complex
mSA2 CAR-T: Monomeric Streptavidin 2-based Chimeric Antigen Receptor T-cells
MV-CEA: Measles Virus-based Carcinoembryonic Antigen-expressing Oncolytic Virus
MYXV: Myxoma Virus
NaV: Nanoshield Vaccine
nGBM: Newly Diagnosed Glioblastoma
NO: Nitric Oxide
nsIL12: Non-secreting Interleukin-12
OA@Cho: Cholesterol-modified Adenovirus
oHSV-1: Oncolytic Herpes Simplex Virus-1
OS: Overall Survival
PAMPs: Pathogen-associated molecular patterns
PBMCs: Peripheral blood mononuclear cells
PDT: Photodynamic Therapy
PFS: Progression-Free Survival
PPV: Personalized Peptide Vaccine
PVSRIPO: Poliovirus (Recombinant Nonpathogenic Poliovirus: Rhinovirus Recombinant)
rGBM: Recurrent Glioblastoma
ROS: Reactive Oxygen Species
scFv: Single-chain variable fragment
SPOD-NV: Survivin Peptide-CpG Nanovaccine
Stars NV: Sonodynamic Nanovaccine
STAT: Signal Transducer and Activator of Transcription
SurVaxM: Survivin-targeting Peptide Vaccine
TAAs: Tumor-associated Antigens
TAMs: Tumor-associated Macrophages
TCF: Tumor Cavity Fluid
TCR-T: T Cell Receptor-engineered T Cells
TERT: Telomerase Reverse Transcriptase
TGFB2: Transforming Growth Factor Beta 2
TGFβ: Transforming Growth Factor Beta
TILs: Tumor-infiltrating Lymphocytes
TME: Tumor microenvironment
TMZ: Temozolomide
Tregs: Regulatory T Cells
TSAs: Tumor-specific Antigens
WHO: World Health Organization
WT1: Wilms Tumor 1
ZIKV: Zika Virus
ZNF638: Zinc Finger Protein 638

## References

[1] Fekrirad Z, Barzegar Behrooz A, Ghaemi S, Khosrojerdi A, Zarepour A, Zarrabi A (2022). Immunology Meets Bioengineering: Improving the Effectiveness of Glioblastoma Immunotherapy. Cancers (Basel).

[2] Louis DN, Perry A, Wesseling P, Brat DJ, Cree IA, Figarella-Branger D (2021). The 2021 WHO Classification of Tumors of the Central Nervous System: a summary. Neuro Oncol.

[3] Komori T (2022). Grading of adult diffuse gliomas according to the 2021 WHO Classification of Tumors of the Central Nervous System. Lab Invest.

[4] Craig H, Tamar B, Roger J P, Patrick Y W (2022). Clinical implications of the 2021 edition of the WHO classification of central nervous system tumours. Nat Rev Neurol.

[5] Choudhary N, Osorio RC, Oh JY, Aghi MK (2023). Metabolic Barriers to Glioblastoma Immunotherapy. Cancers (Basel).

[6] Yasinjan F, Xing Y, Geng H, Guo R, Yang L, Liu Z (2023). Immunotherapy: a promising approach for glioma treatment. Front Immunol.

[7] Zhou Y, Shi F, Zhu J, Yuan Y (2025). An update on the clinical trial research of immunotherapy for glioblastoma. Front Immunol.

[8] Bianconi A, Palmieri G, Aruta G, Monticelli M, Zeppa P, Tartara F (2023). Updates in Glioblastoma Immunotherapy: An Overview of the Current Clinical and Translational Scenario. Biomedicines.

[9] Gagliardi F, De Domenico P, Snider S, Roncelli F, Comai S, Mortini P (2025). Immunomodulatory mechanisms driving tumor escape in glioblastoma: The central role of IDO and tryptophan metabolism in local and systemic immunotolerance. Crit Rev Oncol Hematol.

[10] Desland FA, Hormigo A (2020). The CNS and the Brain Tumor Microenvironment: Implications for Glioblastoma Immunotherapy. Int J Mol Sci.

[11] Mishchenko TA, Turubanova VD, Gorshkova EN, Krysko O, Vedunova MV, Krysko DV (2023). Glioma: bridging the tumor microenvironment, patient immune profiles and novel personalized immunotherapy. Front Immunol.

[12] Zhao T, Li C, Ge H, Lin Y, Kang D (2022). Glioblastoma vaccine tumor therapy research progress. Chin Neurosurg J.

[13] Bagley SJ, Binder ZA, Lamrani L, Marinari E, Desai AS, Nasrallah MP (2024). Repeated peripheral infusions of anti-EGFRvIII CAR T cells in combination with pembrolizumab show no efficacy in glioblastoma: a phase 1 trial. Nat Cancer.

[14] O'Rourke DM, Nasrallah MP, Desai A, Melenhorst JJ, Mansfield K, Morrissette JJD (2017). A single dose of peripherally infused EGFRvIII-directed CAR T cells mediates antigen loss and induces adaptive resistance in patients with recurrent glioblastoma. Sci Transl Med.

[15] Bagley SJ, Logun M, Fraietta JA, Wang X, Desai AS, Bagley LJ (2024). Intrathecal bivalent CAR T cells targeting EGFR and IL13Rα2 in recurrent glioblastoma: phase 1 trial interim results. Nat Med.

[16] Sloan AE, Dansey R, Zamorano L, Barger G, Hamm C, Diaz F (2000). Adoptive immunotherapy in patients with recurrent malignant glioma: preliminary results of using autologous whole-tumor vaccine plus granulocyte-macrophage colony-stimulating factor and adoptive transfer of anti-CD3-activated lymphocytes. Neurosurg Focus.

[17] Yamanaka R, Abe T, Yajima N, Tsuchiya N, Homma J, Kobayashi T (2003). Vaccination of recurrent glioma patients with tumour lysate-pulsed dendritic cells elicits immune responses: results of a clinical phase I/II trial. Br J Cancer.

[18] Izumoto S (2012). Peptide vaccine. Adv Exp Med Biol.

[19] Rampling R, Peoples S, Mulholland PJ, James A, Al-Salihi O, Twelves CJ (2016). A Cancer Research UK First Time in Human Phase I Trial of IMA950 (Novel Multipeptide Therapeutic Vaccine) in Patients with Newly Diagnosed Glioblastoma. Clin Cancer Res.

[20] Zelba H, Shao B, Rabsteyn A, Reinhardt A, Greve C, Oenning L (2025). In-depth characterization of vaccine-induced neoantigen-specific T cells in patients with IDH1-mutant glioma undergoing personalized peptide vaccination. J Immunother Cancer.

[21] Berneman ZN, De Laere M, Germonpré P, Huizing MT, Willemen Y, Lion E (2025). WT1-mRNA dendritic cell vaccination of patients with glioblastoma multiforme, malignant pleural mesothelioma, metastatic breast cancer, and other solid tumors: type 1 T-lymphocyte responses are associated with clinical outcome. J Hematol Oncol.

[22] Ridolfi L, Gurrieri L, Riva N, Bulgarelli J, De Rosa F, Guidoboni M (2024). First step results from a phase II study of a dendritic cell vaccine in glioblastoma patients (CombiG-vax). Front Immunol.

[23] Bota DA, Piccioni DE, Duma CM, Kesari S, Carrillo JA, LaRocca RV (2025). Phase 2 trial of personal dendritic cell vaccines in newly diagnosed glioblastoma: 3-year follow-up and correlations with survival. Hum Vaccin Immunother.

[24] Wang QT, Nie Y, Sun SN, Lin T, Han RJ, Jiang J (2020). Tumor-associated antigen-based personalized dendritic cell vaccine in solid tumor patients. Cancer Immunol Immunother.

[25] Wen PY, Reardon DA, Armstrong TS, Phuphanich S, Aiken RD, Landolfi JC (2019). A Randomized Double-Blind Placebo-Controlled Phase II Trial of Dendritic Cell Vaccine ICT-107 in Newly Diagnosed Patients with Glioblastoma. Clin Cancer Res.

[26] Yao Y, Luo F, Tang C, Chen D, Qin Z, Hua W (2018). Molecular subgroups and B7-H4 expression levels predict responses to dendritic cell vaccines in glioblastoma: an exploratory randomized phase II clinical trial. Cancer Immunol Immunother.

[27] Reap EA, Suryadevara CM, Batich KA, Sanchez-Perez L, Archer GE, Schmittling RJ (2018). Dendritic Cells Enhance Polyfunctionality of Adoptively Transferred T Cells That Target Cytomegalovirus in Glioblastoma. Cancer Res.

[28] Mitchell DA, Batich KA, Gunn MD, Huang MN, Sanchez-Perez L, Nair SK (2015). Tetanus toxoid and CCL3 improve dendritic cell vaccines in mice and glioblastoma patients. Nature.

[29] Batich KA, Reap EA, Archer GE, Sanchez-Perez L, Nair SK, Schmittling RJ (2017). Long-term Survival in Glioblastoma with Cytomegalovirus pp65-Targeted Vaccination. Clin Cancer Res.

[30] Liau LM, Ashkan K, Tran DD, Campian JL, Trusheim JE, Cobbs CS (2018). First results on survival from a large Phase 3 clinical trial of an autologous dendritic cell vaccine in newly diagnosed glioblastoma. J Transl Med.

[31] Weller M, Butowski N, Tran DD, Recht LD, Lim M, Hirte H (2017). Rindopepimut with temozolomide for patients with newly diagnosed, EGFRvIII-expressing glioblastoma (ACT IV): a randomised, double-blind, international phase 3 trial. Lancet Oncol.

[32] Schuster J, Lai RK, Recht LD, Reardon DA, Paleologos NA, Groves MD (2015). A phase II, multicenter trial of rindopepimut (CDX-110) in newly diagnosed glioblastoma: the ACT III study. Neuro Oncol.

[33] Platten M, Bunse L, Wick A, Bunse T, Le Cornet L, Harting I (2021). A vaccine targeting mutant IDH1 in newly diagnosed glioma. Nature.

[34] Ahluwalia MS, Reardon DA, Abad AP, Curry WT, Wong ET, Figel SA (2023). Phase IIa Study of SurVaxM Plus Adjuvant Temozolomide for Newly Diagnosed Glioblastoma. J Clin Oncol.

[35] Withers HG, Figel SA, Qiu J, Ahluwalia MS, Reardon D, Abad AP (2025). Intratumoral B cell and interferon signatures in newly diagnosed glioblastoma are associated with longer survival in patients treated with SurVaxM. Cancer Immunol Immunother.

[36] Fu S, Piccioni DE, Liu H, Lukas RV, Kesari S, Aregawi D (2021). A phase I study of the WT2725 dosing emulsion in patients with advanced malignancies. Sci Rep.

[37] Migliorini D, Dutoit V, Allard M, Grandjean Hallez N, Marinari E, Widmer V (2019). Phase I/II trial testing safety and immunogenicity of the multipeptide IMA950/poly-ICLC vaccine in newly diagnosed adult malignant astrocytoma patients. Neuro Oncol.

[38] Tsuboi A, Hashimoto N, Fujiki F, Morimoto S, Kagawa N, Nakajima H (2019). A phase I clinical study of a cocktail vaccine of Wilms' tumor 1 (WT1) HLA class I and II peptides for recurrent malignant glioma. Cancer Immunol Immunother.

[39] Tamura R, Morimoto Y, Kosugi K, Sato M, Oishi Y, Ueda R (2020). Clinical and histopathological analyses of VEGF receptors peptide vaccine in patients with primary glioblastoma - a case series. BMC Cancer.

[40] Okada H, Butterfield LH, Hamilton RL, Hoji A, Sakaki M, Ahn BJ (2015). Induction of robust type-I CD8+ T-cell responses in WHO grade 2 low-grade glioma patients receiving peptide-based vaccines in combination with poly-ICLC. Clin Cancer Res.

[41] Fenstermaker RA, Ciesielski MJ, Qiu J, Yang N, Frank CL, Lee KP (2016). Clinical study of a survivin long peptide vaccine (SurVaxM) in patients with recurrent malignant glioma. Cancer Immunol Immunother.

[42] Sloan AE, Winter K, Gilbert MR, Aldape K, Choi S, Wen PY (2024). NRG-BN002: Phase I study of ipilimumab, nivolumab, and the combination in patients with newly diagnosed glioblastoma. Neuro Oncol.

[43] Lassman AB, Polley MC, Iwamoto FM, Sloan AE, Wang TJC, Aldape KD (2025). Dual Immune Check Point Blockade in MGMT-Unmethylated Newly Diagnosed Glioblastoma: NRG Oncology BN007, a Randomized Phase II/III Clinical Trial. J Clin Oncol.

[44] Narita Y, Arakawa Y, Yamasaki F, Nishikawa R, Aoki T, Kanamori M (2019). A randomized, double-blind, phase III trial of personalized peptide vaccination for recurrent glioblastoma. Neuro Oncol.

[45] Hilf N, Kuttruff-Coqui S, Frenzel K, Bukur V, Stevanović S, Gouttefangeas C (2019). Actively personalized vaccination trial for newly diagnosed glioblastoma. Nature.

[46] Muragaki Y, Ishikawa E, Maruyama T, Nitta M, Saito T, Ikuta S (2023). A multicenter, randomized, placebo-controlled phase IIb trial of an autologous formalin-fixed tumor vaccine for newly diagnosed glioblastomas. J Neurosurg.

[47] Wang Y, Li G, Su J, Liu Y, Zhang X, Zhang G (2025). Tumor-Associated Macrophages Nano-Reprogrammers Induce "Gear Effect" to Empower Glioblastoma Immunotherapy. Small.

[48] Lutz J, Feist RK, Sonntag T, Peguero-Sánchez E, Wolter K, Bick R (2025). Preclinical development of an mRNA-based multiepitope immunotherapeutic for glioblastoma. Cancer Immunol Immunother.

[49] Shi Y, Sun Y, Zhao S, Sun Z, Xia M, Zhong Z (2025). Intranasal and Intravenous Sequential Administration of Survivin Peptide-CpG Nanovaccines Elicits Potent Immunity Toward Glioblastoma. Adv Mater.

[50] Wang C, Feng W, Li J, Wang J, Liu L, Ye SH (2025). Enhanced Nano-Vaccine Utilizing Biomineralized Virus-like Particles for Efficient Glioblastoma Immunotherapy via the Nose-To-Brain Delivery Pathway. ACS Nano.

[51] Jiang S, Lu Z (2025). mRNA-LNP vaccines: rational design, delivery optimization, and clinical translation. J Mater Chem B.

[52] Fang RH, Gao W, Zhang L (2023). Targeting drugs to tumours using cell membrane-coated nanoparticles. Nat Rev Clin Oncol.

[53] Kuang L, Han M, Wu X, Deng Z, Liu T, Yin Y (2025). Starting the Engine and Releasing the Brakes of T-Cell Responses: A Biomimetic Dendritic Cell Nanoplatform for Improved Glioblastoma Immunotherapy. ACS Nano.

[54] Wang W, Zou C, Liu X, He L, Cao Z, Zhu M (2024). Biomimetic Dendritic Cell-Based Nanovaccines for Reprogramming the Immune Microenvironment to Boost Tumor Immunotherapy. ACS Nano.

[55] Cheng W, Yang J, Pan Y, Qu H, Duan Z, Wu J (2025). Noninvasive Activation of Local and Systemic Immunity with a Sequential-Targeting Sonodynamic Nanovaccine to Treat Glioblastoma. ACS Nano.

[56] Brüßeler MMT, Zam A, Moreno-Zafra VM, Rouatbi N, Hassuneh OWM, Marrocu A (2024). Polyinosinic/Polycytidylic Lipid Nanoparticles Enhance Immune Cell Infiltration and Improve Survival in the Glioblastoma Mouse Model. Mol Pharm.

[57] Yin Q, Li J, Zhang J, Leng J, Zhang K, Gao X (2025). Nanoshield Architecture Harnessing Neoantigen-Targeting Peptides Enables Durable Post-surgical Glioma Immunotherapy. Nano Lett.

[58] Tülümen D, Aydemir E, Ayaz F (2025). Immunoinformatics-based multi-epitope vaccine design using transforming growth factor beta-2 proprotein (TGFB2) for glioblastoma multiforme (GBM): GVac. Methods.

[59] Mirzaie S, Yuan KD, Ni H, Wu XY (2025). Design of a novel multiepitope vaccine against glioblastoma by in silico approaches. Sci Rep.

[60] Kosianova A, Pak O, Zaitsev S, Smirnova P, Bryukhovetskiy I (2025). Clofazimine enhances anti-glioma effect of immunotherapy. Int Immunopharmacol.

[61] Zhang X, Zhou W, Yu J, Jiang R, Han J, Li H (2025). Reprogramming Tumor-Associated Macrophage by Ornithine Decarboxylase Inhibitor and Immune Checkpoint for Orthotopic Glioblastoma Photothermal Immunotherapy. ACS Appl Mater Interfaces.

[62] Puleo J, Polyak K (2022). A Darwinian perspective on tumor immune evasion. Biochim Biophys Acta Rev Cancer.

[63] Ishida Y, Agata Y, Shibahara K, Honjo T (1992). Induced expression of PD-1, a novel member of the immunoglobulin gene superfamily, upon programmed cell death. Embo j.

[64] Brunet JF, Denizot F, Luciani MF, Roux-Dosseto M, Suzan M, Mattei MG (1987). A new member of the immunoglobulin superfamily-CTLA-4. Nature.

[65] Leach DR, Krummel MF, Allison JP (1996). Enhancement of antitumor immunity by CTLA-4 blockade. Science.

[66] Omuro A, Vlahovic G, Lim M, Sahebjam S, Baehring J, Cloughesy T (2018). Nivolumab with or without ipilimumab in patients with recurrent glioblastoma: results from exploratory phase I cohorts of CheckMate 143. Neuro Oncol.

[67] Reardon DA, Brandes AA, Omuro A, Mulholland P, Lim M, Wick A (2020). Effect of Nivolumab vs Bevacizumab in Patients With Recurrent Glioblastoma: The CheckMate 143 Phase 3 Randomized Clinical Trial. JAMA Oncol.

[68] Kesari S, Wojcinski A, Pabla S, Seager RJ, Gill JM, Carrillo JA (2024). Pre-radiation Nivolumab plus ipilimumab in patients with newly diagnosed high-grade gliomas. Oncoimmunology.

[69] McFaline-Figueroa JR, Sun L, Youssef GC, Huang R, Li G, Kim J (2024). Neoadjuvant anti-PD1 immunotherapy for surgically accessible recurrent glioblastoma: clinical and molecular outcomes of a stage 2 single-arm expansion cohort. Nat Commun.

[70] Weathers SP, Li X, Zhu H, Damania AV, Knafl M, McKinley B (2025). Improved overall survival in an anti-PD-L1 treated cohort of newly diagnosed glioblastoma patients is associated with distinct immune, mutation, and gut microbiome features: a single arm prospective phase I/II trial. Nat Commun.

[71] Louis DN, Perry A, Reifenberger G, von Deimling A, Figarella-Branger D, Cavenee WK (2016). The 2016 World Health Organization Classification of Tumors of the Central Nervous System: a summary. Acta Neuropathol.

[72] Wang D, Zhang J, Bu C, Liu G, Guo G, Zhang Z (2024). Dynamics of tumor *in situ* fluid circulating tumor DNA in recurrent glioblastomas forecasts treatment efficacy of immune checkpoint blockade coupled with low-dose bevacizumab. J Cancer Res Clin Oncol.

[73] Guo G, Zhang Z, Zhang J, Wang D, Xu S, Liu G (2024). Predicting recurrent glioblastoma clinical outcome to immune checkpoint inhibition and low-dose bevacizumab with tumor *in situ* fluid circulating tumor DNA analysis. Cancer Immunol Immunother.

[74] Omuro A, Brandes AA, Carpentier AF, Idbaih A, Reardon DA, Cloughesy T (2023). Radiotherapy combined with nivolumab or temozolomide for newly diagnosed glioblastoma with unmethylated MGMT promoter: An international randomized phase III trial. Neuro Oncol.

[75] Lim M, Weller M, Idbaih A, Steinbach J, Finocchiaro G, Raval RR (2022). Phase III trial of chemoradiotherapy with temozolomide plus nivolumab or placebo for newly diagnosed glioblastoma with methylated MGMT promoter. Neuro Oncol.

[76] Pol JG, Lizarralde-Guerrero M, Checcoli A, Kroemer G (2024). Targeted opening of the blood-brain barrier facilitates doxorubicin/anti-PD-1-based chemoimmunotherapy of glioblastoma. Oncoimmunology.

[77] Lin SW, Yu CP, Tsai JC, Shyong YJ (2024). Delivery of extracellular vesicles loaded with immune checkpoint inhibitors for immunotherapeutic management of glioma. Mater Today Bio.

[78] Zhang Z, Xu X, Du J, Chen X, Xue Y, Zhang J (2024). Redox-responsive polymer micelles co-encapsulating immune checkpoint inhibitors and chemotherapeutic agents for glioblastoma therapy. Nat Commun.

[79] Aytekin E, Maksoud S, Tannous BA, Pehlivan SB, Badr CE (2025). Dual-action nanotherapy: Temozolomide-loaded, anti-PD-L1 scFv-functionalized lipid nanocarriers for targeted glioblastoma therapy. Eur J Pharm Sci.

[80] Chaudhari AP, Budayr OM, Bonacquisti EE, Kussatz CC, Bannon MS, Law KJ (2026). The status of extracellular vesicles as drug carriers and therapeutics. Nature Reviews Bioengineering.

[81] Liu C, Gong S, Du X, Liu Y (2022). Micelle morphology phase diagram in a phospholipid, PEGylated lipid, and peptide amphiphiles ternary system. Chemical Engineering Research and Design.

[82] Fesenmeier DJ, Park S, Kim S, Won YY (2022). Surface mechanical behavior of water-spread poly(styrene)-poly(ethylene glycol) (PS-PEG) micelles at the air-water interface: Effect of micelle size and polymer end/linking group chemistry. J Colloid Interface Sci.

[83] Abdul Rub M, Shafi Sheikh M, Khan F, Azum N, Alghamdi YG, Asiri AM (2021). Impact on micellization between promethazine hydrochloride and ester bonded gemini surfactant in distinct solvents: A multi-faceted procedure. Journal of Molecular Liquids.

[84] Klika Škopić M, Gramse C, Oliva R, Pospich S, Neukirch L, Manisegaran M (2021). Towards DNA-Encoded Micellar Chemistry: DNA-Micelle Association and Environment Sensitivity of Catalysis. Chemistry - A European Journal.

[85] Lang X, Thumu U, Yuan L, Zheng C, Zhang H, He L (2021). Chemical fuel-driven transient polymeric micelle nanoreactors toward reversible trapping and reaction acceleration. Chem Commun (Camb).

[86] Xu Z, Xie Y, Chen W, Deng W (2025). Nanocarrier-Based Systems for Targeted Delivery: Current Challenges and Future Directions. MedComm (2020).

[87] De Martino M, Daviaud C, Lira MC, Hernandez-Zirofsky K, Vanpouille-Box C (2025). Dual blockade of PD-1 and CTLA-4 generates long-lasting immunity against irradiated glioblastoma. Cancer Lett.

[88] Ahn M, Na Y, Choi H, Lee S, Lee J, Park SA (2025). Photoimmuno-Lure Nanoplatform for Enhancing T Cell Expansion in Glioblastoma via Synergistic Treatment of Photodynamic Therapy and Immune Checkpoint Inhibition. Adv Healthc Mater.

[89] Strassheimer F, Elleringmann P, Ludmirski G, Roller B, Macas J, Alekseeva T (2025). CAR-NK cell therapy combined with checkpoint inhibition induces an NKT cell response in glioblastoma. Br J Cancer.

[90] Hou D, Wang S, Castro BA, Katz JL, Dapash M, Arrieta VA (2025). Dual aVß8 Integrin and PD-1 Blockade Overcomes TGFβ-Mediated B-Cell Suppression to Enhance Anti-Tumor Immunity. Neuro Oncol.

[91] Seetharam D, Chandar J, Ramsoomair CK, Desgraves JF, Alvarado Medina A, Hudson AJ (2025). Activating antiviral immune responses potentiates immune checkpoint inhibition in glioblastoma models. J Clin Invest.

[92] Liu T, Sun T, Chen X, Wu J, Sun X, Liu X (2025). Targeting ARPC1B Overcomes Immune Checkpoint Inhibitor Resistance in Glioblastoma by Reversing Protumorigenic Macrophage Polarization. Cancer Res.

[93] Lee J, Kang Y, Lee H, Saravanakumar G, Park SA, Ahn S (2025). Amplifying glioblastoma immunotherapy: T cell shielding through Nitric oxide/reactive oxygen species scavenging nanoparticles Potentiates anti-PD-1. Biomaterials.

[94] Królikowska A, Tarnowski M (2025). CAR-T cells immunotherapy in the treatment of glioblastoma. Cancer Immunol Immunother.

[95] Nagai M (1991). [Advances of BRM therapy of malignant brain tumors]. Gan To Kagaku Ryoho.

[96] Jacobs SK, Wilson DJ, Kornblith PL, Grimm EA (1986). Interleukin-2 and autologous lymphokine-activated killer cells in the treatment of malignant glioma. Preliminary report. J Neurosurg.

[97] Boiardi A, Silvani A, Ruffini PA, Rivoltini L, Parmiani G, Broggi G (1994). Loco-regional immunotherapy with recombinant interleukin-2 and adherent lymphokine-activated killer cells (A-LAK) in recurrent glioblastoma patients. Cancer Immunol Immunother.

[98] Sankhla SK, Nadkarni JS, Bhagwati SN (1996). Adoptive immunotherapy using lymphokine-activated killer (LAK) cells and interleukin-2 for recurrent malignant primary brain tumors. J Neurooncol.

[99] Lin Y, Okada H (2016). Cellular immunotherapy for malignant gliomas. Expert Opin Biol Ther.

[100] Arruda LCM, Karbach J, Kiselicki D, Altmannsberger HM, Sinelnikov E, Gustavus D (2025). Tumor-infiltrating lymphocytes-derived CD8(+) clonotypes infiltrate the tumor tissue and mediate tumor regression in glioblastoma. Oncoimmunology.

[101] Cherkassky L, Morello A, Villena-Vargas J, Feng Y, Dimitrov DS, Jones DR (2016). Human CAR T cells with cell-intrinsic PD-1 checkpoint blockade resist tumor-mediated inhibition. J Clin Invest.

[102] Chheda ZS, Kohanbash G, Okada K, Jahan N, Sidney J, Pecoraro M (2018). Novel and shared neoantigen derived from histone 3 variant H3.3K27M mutation for glioma T cell therapy. J Exp Med.

[103] Walton CM, Bell M, O'Neil R, Sahin O, Choi BD, Fecci PE (2025). Chimeric antigen receptor (CAR) T-cell therapy for glioblastoma (GBM): current clinical insights, challenges, and future directions. J Immunother Cancer.

[104] Weathers SP, Penas-Prado M, Pei BL, Ling X, Kassab C, Banerjee P (2020). Glioblastoma-mediated Immune Dysfunction Limits CMV-specific T Cells and Therapeutic Responses: Results from a Phase I/II Trial. Clin Cancer Res.

[105] Lim J, Park Y, Ahn JW, Sim J, Kang SJ, Hwang S (2021). Autologous adoptive immune-cell therapy elicited a durable response with enhanced immune reaction signatures in patients with recurrent glioblastoma: An open label, phase I/IIa trial. PLoS One.

[106] Jaramillo M, Sarfraz Z, Ganiyani MA, Mustafayev FNA, Qidwai K, Mustafayev K (2025). CTIM-26. TOXICITY PROFILE OF CAR-T CELL THERAPY IN GLIOBLASTOMA: A META-ANALYSIS OF CYTOKINE RELEASE SYNDROME, NEUROTOXICITY, AND ADVERSE EVENTS. Neuro-Oncology.

[107] Geraghty AC, Acosta-Alvarez L, Rotiroti MC, Dutton S, O'Dea MR, Kim W (2025). Immunotherapy-related cognitive impairment after CAR T cell therapy in mice. Cell.

[108] Graham CE, Velasco R, Alarcon Tomas A, Stewart OP, Dachy G, del Bufalo F (2025). Non-ICANS neurological complications after CAR T-cell therapies: recommendations from the EBMT Practice Harmonisation and Guidelines Committee. The Lancet Oncology.

[109] Karschnia P, Dietrich J (2025). Neurological complications of CAR T cell therapy for cancers. Nature Reviews Neurology.

[110] Bagley SJ, Desai AS, Fraietta JA, Silverbush D, Chafamo D, Freeburg NF (2025). Intracerebroventricular bivalent CAR T cells targeting EGFR and IL-13Rα2 in recurrent glioblastoma: a phase 1 trial. Nat Med.

[111] Brown CE, Badie B, Barish ME, Weng L, Ostberg JR, Chang WC (2015). Bioactivity and Safety of IL13Rα2-Redirected Chimeric Antigen Receptor CD8+ T Cells in Patients with Recurrent Glioblastoma. Clin Cancer Res.

[112] Brown CE, Rodriguez A, Palmer J, Ostberg JR, Naranjo A, Wagner JR (2022). Off-the-shelf, steroid-resistant, IL13Rα2-specific CAR T cells for treatment of glioblastoma. Neuro Oncol.

[113] Liu Z, Zhou J, Yang X, Liu Y, Zou C, Lv W (2023). Safety and antitumor activity of GD2-Specific 4SCAR-T cells in patients with glioblastoma. Mol Cancer.

[114] Barish ME, Aftabizadeh M, Hibbard J, Blanchard MS, Ostberg JR, Wagner JR (2025). Chlorotoxin-directed CAR T cell therapy for recurrent glioblastoma: Interim clinical experience demonstrating feasibility and safety. Cell Rep Med.

[115] Kourtesakis A, Bailey E, Chow HNH, Rohdjeß H, Mussnig N, Agardy DA (2025). Utilization of universal-targeting mSA2 CAR-T cells for the treatment of glioblastoma. Oncoimmunology.

[116] Zhai Y, Li G, Pan C, Yu M, Hu H, Wang D (2025). The development and potent antitumor efficacy of CD44/CD133 dual-targeting IL7Rα-armored CAR-T cells against glioblastoma. Cancer Lett.

[117] Liu L, He P, Wang Y, Ma F, Li D, Bai Z (2025). Engineering sonogenetic EchoBack-CAR T cells. Cell.

[118] Cook DR, Boesteanu AC, Yin Y, Reid R, Roccograndi L, Dahmane N (2025). Checkpoint antibody receptor modified ARMed CAR T circumvents the suppressive immunome in GBM. Front Immunol.

[119] Liu J, Dai K, Saliu MA, Salisu MD, Gan J, Afolabi LO (2024). Sodium valproate enhances efficacy of NKG2D CAR-T cells against glioblastoma. Front Immunol.

[120] Wu BX, Kreatsoulas D, Cam H, Bolyard C, Chang Y, Mandula J (2025). Targeting TGFβ docking receptor Glycoprotein A Repetitions Predominant (GARP) via novel chimeric antigen receptor (CAR)-T cell platform to treat glioblastoma. Neuro Oncol.

[121] Sætersmoen M, Kotchetkov IS, Torralba-Raga L, Mansilla-Soto J, Sohlberg E, Krokeide SZ (2025). Targeting HLA-E-overexpressing cancers with a NKG2A/C switch receptor. Med.

[122] Foster JB, Madsen PJ, Harvey K, Griffin C, Stern A, Patterson L (2025). Transient mRNA CAR T cells targeting GD2 provide dose-adjusted efficacy against diffuse midline glioma and high-grade glioma models. Neuro Oncol.

[123] Nehama D, Di Ianni N, Musio S, Du H, Patané M, Pollo B (2019). B7-H3-redirected chimeric antigen receptor T cells target glioblastoma and neurospheres. EBioMedicine.

[124] Yin P, Yang J, Jiang Y, Han L, Xiong T, Liu H (2025). Enhanced FOS expression improves tumor clearance and resists exhaustion in NR4A3-deficient CAR T cells under chronic antigen exposure. Sci Adv.

[125] Stemer G, Mittermayr T, Schnell-Inderst P, Wild C (2025). Costs, challenges and opportunities of decentralised chimeric antigen receptor T-cell production: a literature review and clinical experts' interviews. Eur J Hosp Pharm.

[126] Ma HL, Garcia LSA, Ishiba R, Dametto LC, da Silva Moraes KA, Oliveira Ferreira R (2026). Modeling glioblastoma in 3D hydrogels enables investigation of Zika virus targeting and immune modulation in oncolytic virotherapy. Biomater Adv.

[127] Markert JM, Razdan SN, Kuo HC, Cantor A, Knoll A, Karrasch M (2014). A phase 1 trial of oncolytic HSV-1, G207, given in combination with radiation for recurrent GBM demonstrates safety and radiographic responses. Mol Ther.

[128] Kicielinski KP, Chiocca EA, Yu JS, Gill GM, Coffey M, Markert JM (2014). Phase 1 clinical trial of intratumoral reovirus infusion for the treatment of recurrent malignant gliomas in adults. Mol Ther.

[129] Freeman AI, Zakay-Rones Z, Gomori JM, Linetsky E, Rasooly L, Greenbaum E (2006). Phase I/II trial of intravenous NDV-HUJ oncolytic virus in recurrent glioblastoma multiforme. Mol Ther.

[130] Lang FF, Conrad C, Gomez-Manzano C, Yung WKA, Sawaya R, Weinberg JS (2018). Phase I Study of DNX-2401 (Delta-24-RGD) Oncolytic Adenovirus: Replication and Immunotherapeutic Effects in Recurrent Malignant Glioma. J Clin Oncol.

[131] Desjardins A, Gromeier M, Herndon JE 2nd, Beaubier N, Bolognesi DP, Friedman AH (2018). Recurrent Glioblastoma Treated with Recombinant Poliovirus. N Engl J Med.

[132] Nassiri F, Patil V, Yefet LS, Singh O, Liu J, Dang RMA (2023). Oncolytic DNX-2401 virotherapy plus pembrolizumab in recurrent glioblastoma: a phase 1/2 trial. Nat Med.

[133] Tur-Planells V, Bykov Y, Dawodu G, García-Romero N, Izpura-Luis S, Pérez-Rodríguez L (2025). Infectious bursal disease virus (IBDV) as a novel oncolytic virotherapy in glioblastoma. J Immunother Cancer.

[134] Guo C, Long Z, Lin P, Shen Y, Zhong Y, Qian J (2025). BRD9 inhibition overcomes oncolytic virus therapy resistance in glioblastoma. Cell Rep Med.

[135] Sahu U, Mullarkey MP, Murphy SA, Anderson JC, Putluri V, Kamal AHM (2025). IDH status dictates oHSV mediated metabolic reprogramming affecting anti-tumor immunity. Nat Commun.

[136] Galanis E, Dooley KE, Keith Anderson S, Kurokawa CB, Carrero XW, Uhm JH (2024). Carcinoembryonic antigen-expressing oncolytic measles virus derivative in recurrent glioblastoma: a phase 1 trial. Nat Commun.

[137] Ning W, Qian X, Dunmall LC, Liu F, Guo Y, Li S (2024). Non-secreting IL12 expressing oncolytic adenovirus Ad-TD-nsIL12 in recurrent high-grade glioma: a phase I trial. Nat Commun.

[138] Fares J, Ahmed AU, Ulasov IV, Sonabend AM, Miska J, Lee-Chang C (2021). Neural stem cell delivery of an oncolytic adenovirus in newly diagnosed malignant glioma: a first-in-human, phase 1, dose-escalation trial. Lancet Oncol.

[139] Todo T, Ito H, Ino Y, Ohtsu H, Ota Y, Shibahara J (2022). Intratumoral oncolytic herpes virus G47∆ for residual or recurrent glioblastoma: a phase 2 trial. Nat Med.

[140] Todo T, Ino Y, Ohtsu H, Shibahara J, Tanaka M (2022). A phase I/II study of triple-mutated oncolytic herpes virus G47∆ in patients with progressive glioblastoma. Nat Commun.

[141] He Y, Li W, Zhang X, Cui Z (2025). Oncolytic Virus Targeted Therapy for Glioma via Intravenous Delivery. Adv Healthc Mater.

[142] Weller SK, Coen DM (2012). Herpes simplex viruses: mechanisms of DNA replication. Cold Spring Harb Perspect Biol.

[143] Ferreira RS, Jandrey EHF, Granha I, Endo AK, Ferreira RO, Araujo BHS (2024). Differential Replication and Oncolytic Effects of Zika Virus in Aggressive CNS Tumor Cells: Insights from Organoid and Tumoroid Models. Viruses.

[144] Jiang H, Nace R, Ferguson C, Zhang L, Peng KW, Russell SJ (2025). Oncolytic cytomegaloviruses expressing EGFR-retargeted fusogenic glycoprotein complex and drug-controllable interleukin 12. Cell Rep Med.

[145] Jazowiecka-Rakus J, Pogoda-Mieszczak K, Rahman MM, McFadden G, Sochanik A (2024). Adipose-Derived Stem Cells as Carrier of Pro-Apoptotic Oncolytic Myxoma Virus: To Cross the Blood-Brain Barrier and Treat Murine Glioma. Int J Mol Sci.

[146] Tang F, Zhang Z, Xu J, Wang Z, Chen Y, Huang T (2025). A chimeric oncolytic adenovirus carried by macrophages for glioma immunotherapy. Biochem Biophys Res Commun.

[147] Niu A, Lv Y, Chen Y, Liu Y, Luo C, Zheng M (2025). Cholesterol surface-modified oncolytic adenovirus enriched with apolipoprotein E penetrates the blood-brain barrier to target glioblastoma immunotherapy. Mater Today Bio.

[148] Nazarenko AS, Shkirdova AO, Orlova EA, Biryukova YK, Vorovitch MF, Kolyasnikova NM (2024). Viral-Porphyrin Combo: Photodynamic and Oncolytic Viral Therapy for Potent Glioblastoma Treatment. Int J Mol Sci.

[149] Wang S, Wei J (2022). Distinguishing the pros and cons of metabolic reprogramming in oncolytic virus immunotherapy. Int J Cancer.

[150] Chen Y, Bian S, Zhang J, Luan Y, Yin B, Dai W (2024). HSV-1-induced N6-methyladenosine reprogramming via ICP0-mediated suppression of METTL14 potentiates oncolytic activity in glioma. Cell Rep.

[151] Li Y, Qin X, Liang C, Wang L (2026). Progress in the research and development of oncolytic virus therapies. Front Pharmacol.

[152] Quail DF, Joyce JA (2017). The Microenvironmental Landscape of Brain Tumors. Cancer Cell.

[153] Habashy KJ, Mansour R, Moussalem C, Sawaya R, Massaad MJ (2022). Challenges in glioblastoma immunotherapy: mechanisms of resistance and therapeutic approaches to overcome them. British Journal of Cancer.

[154] Sampson JH, Gunn MD, Fecci PE, Ashley DM (2020). Brain immunology and immunotherapy in brain tumours. Nat Rev Cancer.

[155] Pardridge WM (2019). Blood-Brain Barrier and Delivery of Protein and Gene Therapeutics to Brain. Front Aging Neurosci.

[156] Arvanitis CD, Ferraro GB, Jain RK (2020). The blood-brain barrier and blood-tumour barrier in brain tumours and metastases. Nat Rev Cancer.

[157] Koyama S, Akbay EA, Li YY, Herter-Sprie GS, Buczkowski KA, Richards WG (2016). Adaptive resistance to therapeutic PD-1 blockade is associated with upregulation of alternative immune checkpoints. Nat Commun.

[158] Jackson CM, Choi J, Lim M (2019). Mechanisms of immunotherapy resistance: lessons from glioblastoma. Nat Immunol.

[159] Herrera GJ, Sun L, Lee AH, Everson RG, Nathanson DA, Liau LM (2024). 819 Temporal influence of PD-1 blockade after vaccination on macrophage-driven CD8+ T cell exhaustion within the glioblastoma microenvironment. Journal for ImmunoTherapy of Cancer.

[160] Friedrich M, Hahn M, Michel J, Sankowski R, Kilian M, Kehl N (2023). Dysfunctional dendritic cells limit antigen-specific T cell response in glioma. Neuro Oncol.

[161] Raphael I, Kumar R, McCarl LH, Shoger K, Wang L, Sandlesh P (2021). TIGIT and PD-1 Immune Checkpoint Pathways Are Associated With Patient Outcome and Anti-Tumor Immunity in Glioblastoma. Front Immunol.

[162] Losurdo A, Di Muzio A, Cianciotti BC, Dipasquale A, Persico P, Barigazzi C (2024). T Cell Features in Glioblastoma May Guide Therapeutic Strategies to Overcome Microenvironment Immunosuppression. Cancers (Basel).

[163] Lin C, Teng W, Tian Y, Li S, Xia N, Huang C (2024). Immune landscape and response to oncolytic virus-based immunotherapy. Front Med.

[164] Johnson AL, Lopez-Bertoni H (2024). Cellular diversity through space and time: adding new dimensions to GBM therapeutic development. Front Genet.

[165] Brown NF, Carter TJ, Ottaviani D, Mulholland P (2018). Harnessing the immune system in glioblastoma. Br J Cancer.

[166] Dong W, Luo Y, He D, Zhang M, Zeng J, Chen Y (2024). Oncolytic virotherapy against lung cancer: key receptors and signaling pathways of viral entry. Front Immunol.

[167] Kollmann CF, van Montfoort N, Cordelier P, Pol J, Olagnier D (2025). Oncolytic virotherapy: Sparking durable anti-tumor immunity through microenvironment modulation. Semin Immunol.

[168] Fu M, Xue B, Miao X, Gao Z (2025). Overcoming immunotherapy resistance in glioblastoma: challenges and emerging strategies. Front Pharmacol.

[169] Xing Y, Liu C, Feng Y, Li S, Chen Y (2025). Vaccine therapies for glioma: clinical frontiers and potential breakthrough. Front Oncol.

[170] Skadborg SK, Maarup S, Draghi A, Borch A, Hendriksen S, Mundt F (2024). Nivolumab Reaches Brain Lesions in Patients with Recurrent Glioblastoma and Induces T-cell Activity and Upregulation of Checkpoint Pathways. Cancer Immunol Res.

[171] Sari G, Rock KL (2023). Tumor immune evasion through loss of MHC class-I antigen presentation. Curr Opin Immunol.

[172] Badrinath S, Dellacherie MO, Li A, Zheng S, Zhang X, Sobral M (2022). A vaccine targeting resistant tumours by dual T cell plus NK cell attack. Nature.

[173] Xu J, Xia Z, Wang S, Xia Q (2025). Resistance to oncolytic virotherapy: Multidimensional mechanisms and therapeutic breakthroughs (Review). Int J Mol Med.

[174] Ouyang G, Liu Y, Liu J, Huang L, Luo F, Li L (2023). Efficacy and safety of reduced-dose chemotherapy plus immunotherapy in patients with lung squamous cell carcinoma: A real-world observational study. Cancer Med.

[175] Pickl M, Ruge E, Venturi M (2012). Predictive markers in early research and companion diagnostic developments in oncology. N Biotechnol.

[176] Hegi ME, Diserens AC, Gorlia T, Hamou MF, de Tribolet N, Weller M (2005). MGMT gene silencing and benefit from temozolomide in glioblastoma. N Engl J Med.

[177] Ballo MT, Conlon P, Lavy-Shahaf G, Kinzel A, Vymazal J, Rulseh AM (2023). Association of Tumor Treating Fields (TTFields) therapy with survival in newly diagnosed glioblastoma: a systematic review and meta-analysis. J Neurooncol.

[178] Giunco S, Padovan M, Angelini C, Cavallin F, Cerretti G, Morello M (2023). Prognostic role and interaction of TERT promoter status, telomere length and MGMT promoter methylation in newly diagnosed IDH wild-type glioblastoma patients. ESMO Open.

[179] Eckel-Passow JE, Lachance DH, Molinaro AM, Walsh KM, Decker PA, Sicotte H (2015). Glioma Groups Based on 1p/19q, IDH, and TERT Promoter Mutations in Tumors. N Engl J Med.

[180] Vuong HG, Nguyen TQ, Ngo TNM, Nguyen HC, Fung KM, Dunn IF (2020). The interaction between TERT promoter mutation and MGMT promoter methylation on overall survival of glioma patients: a meta-analysis. BMC Cancer.

[181] Stichel D, Ebrahimi A, Reuss D, Schrimpf D, Ono T, Shirahata M (2018). Distribution of EGFR amplification, combined chromosome 7 gain and chromosome 10 loss, and TERT promoter mutation in brain tumors and their potential for the reclassification of IDHwt astrocytoma to glioblastoma. Acta Neuropathol.

[182] Makawi A, Khalafallah SA, Faris IM, Alfaki M (2024). Comprehensive Analysis Reveals Epithelial Growth Factor Receptor as a Potential Diagnostic Biomarker in Glioblastoma Multiforme. Cureus.

[183] Ermoian RP, Furniss CS, Lamborn KR, Basila D, Berger MS, Gottschalk AR (2002). Dysregulation of PTEN and protein kinase B is associated with glioma histology and patient survival. Clin Cancer Res.

[184] Davalan W, Alkins R (2025). Prognostic and predictive determinants in high-grade gliomas: integrating tumor-intrinsic biology with patient and system-level factors. Front Neurol.

[185] Franceschi E, De Biase D, Di Nunno V, Pession A, Tosoni A, Gatto L (2021). IDH1 Non-Canonical Mutations and Survival in Patients with Glioma. Diagnostics (Basel).

[186] Takahashi Y, Nakamura H, Makino K, Hide T, Muta D, Kamada H (2013). Prognostic value of isocitrate dehydrogenase 1, O6-methylguanine-DNA methyltransferase promoter methylation, and 1p19q co-deletion in Japanese malignant glioma patients. World J Surg Oncol.

[187] Hariharan S, Whitfield BT, Pirozzi CJ, Waitkus MS, Brown MC, Bowie ML (2024). Interplay between ATRX and IDH1 mutations governs innate immune responses in diffuse gliomas. Nat Commun.

[188] Haase S, Garcia-Fabiani MB, Carney S, Altshuler D, Núñez FJ, Méndez FM (2018). Mutant ATRX: uncovering a new therapeutic target for glioma. Expert Opin Ther Targets.

[189] Yip S, Butterfield YS, Morozova O, Chittaranjan S, Blough MD, An J (2012). Concurrent CIC mutations, IDH mutations, and 1p/19q loss distinguish oligodendrogliomas from other cancers. J Pathol.

[190] Brat DJ, Verhaak RG, Aldape KD, Yung WK, Salama SR, Cooper LA (2015). Comprehensive, Integrative Genomic Analysis of Diffuse Lower-Grade Gliomas. N Engl J Med.

[191] Appay R, Dehais C, Maurage CA, Alentorn A, Carpentier C, Colin C (2019). CDKN2A homozygous deletion is a strong adverse prognosis factor in diffuse malignant IDH-mutant gliomas. Neuro Oncol.

[192] Mousavi Salehi A, Khavanin A, Azizidoost S, Cheraghzadeh M, Khombi Shooshtari M, Farzaneh M (2026). Targeting XBP1 in Cancer: A Review of Therapeutic Approaches and Strategies. Anticancer Agents Med Chem.

[193] Elias MG, Hadjiyiannis H, Vafaee F, Scott KF, de Souza P, Becker TM (2025). The Quest for Non-Invasive Diagnosis: A Review of Liquid Biopsy in Glioblastoma. Cancers (Basel).

[194] Baldini C, Porte M, Rouleau E, Touat M (2025). Liquid biopsy in gliomas-are we there yet?. Annals of Oncology.

[195] Bae WH, Maraka S, Daher A (2024). Challenges and advances in glioblastoma targeted therapy: the promise of drug repurposing and biomarker exploration. Frontiers in Oncology. 2024; Volume 14 -.

[196] Jo Y, Lee SM, Hong C (2026). From splicing noise to therapeutic signaling: RCAN1-4 as a neoepitope in glioblastoma. Cellular & Molecular Immunology.

[197] Taori S, Habib A, Adida S, Gecici NN, Sharma N, Calcaterra M (2025). Circulating biomarkers in high-grade gliomas: current insights and future perspectives. Journal of Neuro-Oncology.

[198] Qi Y, Hu L, Ji C, Yang X, Yao J, Chen D (2024). B7-H4 reduces the infiltration of CD8+T cells and induces their anti-tumor dysfunction in gliomas. Neoplasia.

[199] Xiao D, Zhang H, Liu Y, Li Y, Li G, Ning Y (2026). Oncolytic viruses: advanced strategies in cancer therapy. Signal Transduct Target Ther.

[200] Alwithenani A, Hengswat P, Chiocca EA (2025). Oncolytic viruses as cancer therapeutics: From mechanistic insights to clinical translation. Mol Ther.

